# An Adenylate Kinase OsAK3 Involves Brassinosteroid Signaling and Grain Length in Rice (*Oryza sativa* L.)

**DOI:** 10.1186/s12284-021-00546-0

**Published:** 2021-12-28

**Authors:** Jiaqi Zhang, Xiuying Gao, Guang Cai, Yuji Wang, Jianbo Li, Huaying Du, Ruqin Wang, Hongsheng Zhang, Ji Huang

**Affiliations:** 1 State Key Laboratory of Crop Genetics and Germplasm Enhancement, College of Agriculture, Nanjing Agricultural University, Nanjing, 210095 China; 2 Jiangsu Provincial Engineering Research Center of Seed Industry Science and Technology, Nanjing, 210095 China

**Keywords:** Grain length, Brassinosteroids, Adenylate kinase, OsAK3, qGL3

## Abstract

**Background:**

Grain size is one of the major determinants of cereal crop yield. As a class of plant polyhydroxysteroids, brassinosteroids (BRs) play essential roles in the regulation of grain size and plant architecture in rice. In a previous research, we cloned *qGL3*/*OsPPKL1* encoding a protein phosphatase with Kelch-like repeat domains, which negatively regulates BR signaling and grain length in rice.

**Results:**

Here, we screened qGL3-interacting proteins (GIPs) via yeast two-hybrid assay and analyzed the phenotypes of the T-DNA insertion mutants of *GIPs*. Among these mutants, mutant *osak3* presents shorter grain length and dwarfing phenotype. *OsAK3* encodes an adenylate kinase, which regulates grain size by controlling cell expansion of rice spikelet glume. Overexpression of *OsAK3* resulted in longer grain length. OsAK3 interacts with qGL3 in vivo and in vitro. Lamina inclination, coleoptile elongation and root inhibition experiments showed that the *osak3* mutant was less sensitive to exogenous brassinolide (BL) treatment. The transcriptional level of *OsAK3* was up-regulated under BL induction. In addition, RNA-Seq data indicate that *OsAK3* is involved in a variety of biological processes that regulate BR signaling and grain development in rice.

**Conclusions:**

Our study reveals a novel BR signaling component OsAK3 in the regulation of grain length, and provides novel clues for uncovering the potential functions of OsAK3 in rice growth and development.

**Supplementary Information:**

The online version contains supplementary material available at 10.1186/s12284-021-00546-0.

## Background

As an important cereal crop, rice is widely planted all over the world. Grain weight is a major determinant of crop yield. Grain size is not only one of the decisive factors of grain weight, but also affects the appearance quality and commodity value of rice (Harberd 2015). Spikelet hull is the dominant factor to limit grain size, and its development is mainly affected by cell expansion and cell proliferation (Li et al. 2018). To date, many genes related to rice grain size have been identified and they involve various signaling pathways, including G protein signaling, the mitogen-activated protein kinase (MAPK) signaling pathway, ubiquitin-mediated proteasome degradation pathway, transcriptional regulation and phytohormone biosynthesis or signaling pathways (Zuo and Li 2014; Li et al. 2018). For example, Gα protein RGA1 and Gβ protein RGB1 from G protein signaling pathway positively regulate rice grain size by affecting cell proliferation. Grain length increases when the Gγ proteins DEP1 and GGC2 bind to Gβ, either alone or together. However, the Gγ protein GS3 reduces grain length via interacting competitively with Gβ (Fan et al. 2006; Utsunomiya et al. 2011; Sun et al. 2018). In MAPK signaling pathway, *O. Sativa* MAPK KINASE4 (OsMKK4) interacts with and phosphorylates OsMAPK6 to control grain size (Liu et al. 2015b). Further biochemical and genetic analysis showed that *O. Sativa* MAPK KINASE KINASE10 (OsMKKK10), OsMKK4 and OsMAPK6 function together to control grain size (Xu et al. 2018). A major QTL locus *GRAIN WIDTH2* (*GW2*) encodes a RING-type E3 ubiquitin ligase controlling the proliferation of spikelet hull and grain width (Song et al. 2007). The deubiquitinating enzyme WTG1/OsOTUB1 controls grain size mainly by affecting cell expansion (Huang et al. 2017). In addition, phytohormone pathways also play important roles in regulating the grain size in rice, such as *O. Sativa* SHORT GRAIN LENGTH (OsSGL) and BIG GRAIN3 (BG3) in cytokinin signaling (Wang et al. 2016; Xiao et al. 2019); BIG GRAIN1 (BG1), SMOS1 and Gnp4/LAX2 in auxin signaling (Aya et al. 2014; Liu et al. 2015a; Zhang et al. 2018b); GRAIN SIZE5 (GS5), SLENDER GRAIN (SLG), GLYCOGEN SYNTHASE KINASE2 (GSK2) and qGL3 in brassinosteroid (BR) signaling (Tong et al. 2012; Xu et al. 2015; Feng et al. 2016; Gao et al. 2019).

BRs are a class of steroid phytohormones that play an important role in multiple processes of plant growth and development, including cell elongation and proliferation, lamina bending, grain filling, stomatal opening, photomorphogenesis and stress responses (Clouse and Sasse 1998; Tong and Chu 2018; Li et al. 2020). In rice, when BRs present, they bind to the receptor complex *O. sativa* BRASSINOSTEROID-INSENSITIVE1 (OsBRI1) and *O. sativa* BRI1-ASSOCIATED RECEPTOR KINASE1 (OsBAK1) (Yamamuro et al. 2000; Li et al. 2009). OsBRI1 phosphorylates *O. sativa* BR-SIGNALING KINASE3 (OsBSK3) to activate BR signal, and the phosphorylated OsBSK3 increases the binding affinity for Arabidopsis *bri1*-SUPPRESSOR1 (AtBSU1) (Zhang et al. 2016). In Arabidopsis, BSU1 plays a positive role in BR signaling, while in rice, its homolog qGL3/OsPPKL1 plays a negative role by mediating the phosphorylation status and stability of protein kinase OsGSK3 and transcription factor (TF) *O. sativa* BRASSINAZOLE RESISTANT1 (OsBZR1) (Gao et al. 2019). In addition to OsBZR1, there are other TFs involving BR signaling, including DWARF AND LOW-TILLERING (DLT), SMOS1/RLA1, OsGRF4, LEAF AND TILLER ANGLE INCREASED CONTROLLER (LIC) and OVATE FAMILY PROTEIN1 (OFP1), OFP3, OFP8 and OFP19 (Tong et al. 2012; Zhang et al. 2012a; Che et al. 2015; Yang et al. 2016, 2018; Qiao et al. 2017; Xiao et al. 2017, 2020). Most of these TFs interact with and are regulated by GSK3-like kinases in BR signaling.

Adenylate Kinases (AKs; EC 2.7.4.3), also known as myokinases, a kind of highly conserved nucleoside monophosphate kinases widely existing in various organisms (Zhang et al. 2018a). This enzyme participates in maintaining nucleotide balance and energy metabolism by catalyzing the interconversion of adenine nucleotides as follows: AMP + ATP ↔ 2ADP (Dzeja and Terzic 2009). There are nine isoenzymes of AKs described in human, and they play a central role in different intracellular compartments (Panayiotou et al. 2014). AK1, AK5, AK7 and AK8 were located in cytosol (Panayiotou et al. 2011). AK2, AK3 and AK4 are mitochondrial isoenzymes, but AK2 was found in the mitochondrial intermembrane space, and the other two isoenzymes were identified in the mitochondrial matrix (Noma et al. 2001). In addition, AK3 is a GTP: AMP phosphotransferase. Unlike other enzymes, its substrate is not ATP, but GTP. AK6 is located in nucleus (Ren et al. 2005), and AK9 is located in both cytoplasm and nucleus (Amiri et al. 2013). In *Arabidopsis thaliana*, ADENYLATE KINASE 6 (AAK6) was identified as an orthologue of human AK6 isoform AD-004 and it’s also located in the nucleus. The *aak6* mutant repressed stem growth compared with wild-type plants, indicating that AAK6 is an essential stem growth factor (Feng et al. 2012). The loss-of-function of *AAK6* leads to the accumulation of 80S ribosomes, thereby affects the cell proliferation and cell size homeostasis in Arabidopsis root growth (Slovak et al. 2020). The absence of Arabidopsis *PLASTIDIAL ADENYLATE KINASE 1* (*AtPADK1,* also known as *AMK1*) increases the photosynthetic amino acid biosynthesis and promotes plant growth (Carrari et al. 2005). Analysis of co-response in Arabidopsis showed that *ADENOSINE MONOPHOSPHATE KINASE 2* (*AMK2*) and *AMK5* expression levels are positively correlated with the expression of photosynthesis and major carbohydrate metabolism genes, while *AMK3*, *AMK4* and *AMK1* are negatively correlated with photosynthesis genes and not related to carbohydrate metabolism genes (Lange et al. 2008). OsAK1 is the closest homolog of Arabidopsis AMK2. OsAK1 located in chloroplast and silencing *OsAK1* in *Epi-ak1* led to a phenotype with albinism of young leaves and panicles along with abnormal chloroplast structure (Wei et al. 2017).

To understand the qGL3-mediated BR signaling, we performed a yeast two-hybrid (Y2H) assay to screen qGL3-interacting proteins (GIPs). Among these T-DNA insertion mutants of *GIPs*, *osak3* exhibited the BR repressed phenotype. In this work, we revealed that *OsAK3*, encoding an adenylate kinase (Kawai et al. 1992), is a novel regulator of rice BR signaling.

## Results

### OsAK3 Physically Interacts with qGL3

**Fig. 1:**
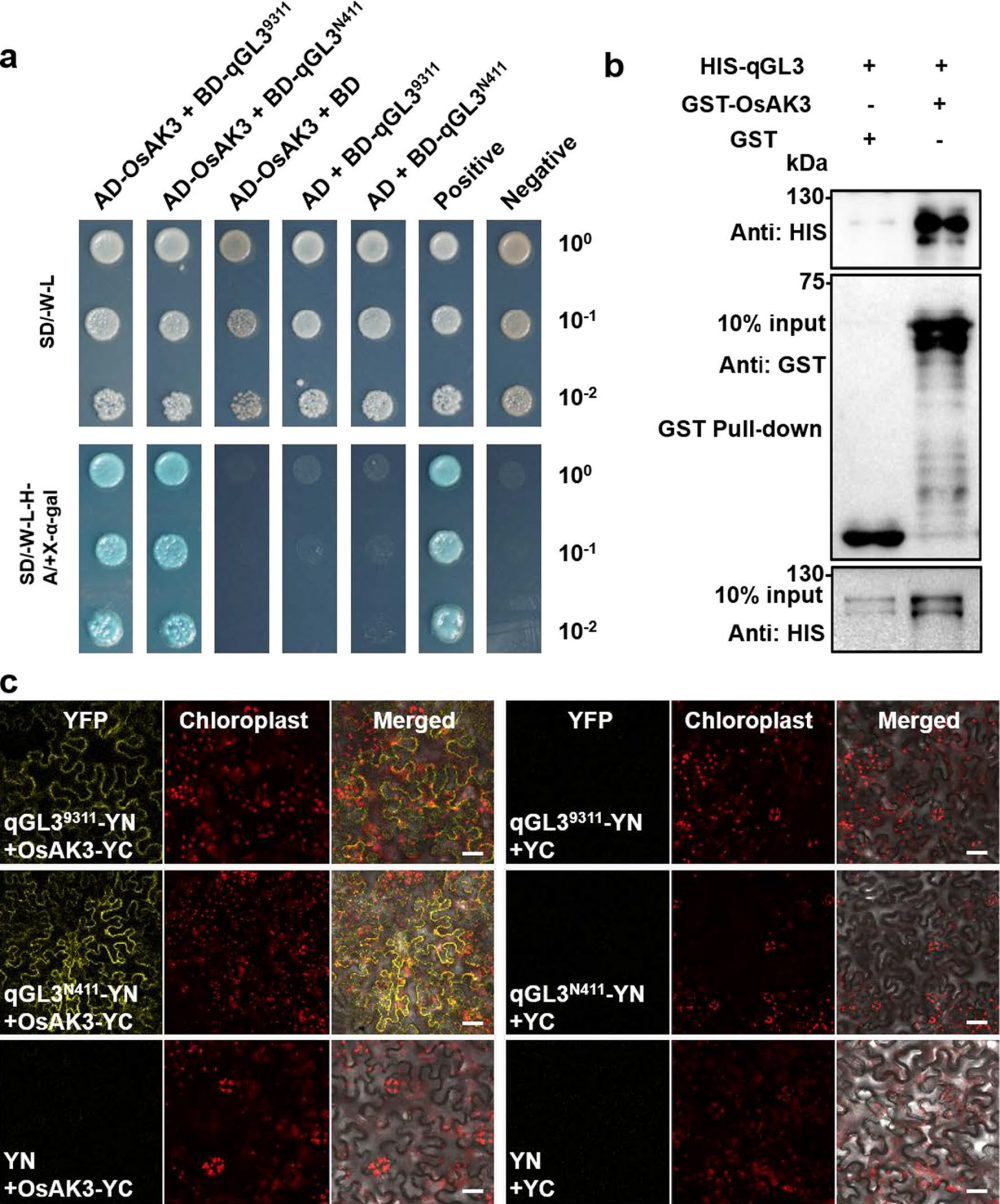
OsAK3 physically interacts with qGL3. **a** Interaction between OsAK3 and qGL3 in Y2H analysis. **b** Interaction between OsAK3 and qGL3 in GST pull-down assay. Immunoblots were probed with anti-GST and anti-His respectively. **c** BiFC analysis of the interaction between OsAK3 and qGL3 (scale bars, 50 μm)

We screened the qGL3-interacting proteins via yeast two-hybrid and identify a rice adenylate kinase (OsAK3) for further analysis (Fig. 1a). Compared with qGL3 from the rice cultivar 9311, qGL3 from the N411 variety is unable to dephosphorylate OsGSK3 due to amino acid changes in its Kelch domain (D364E) (Zhang et al. 2012b; Gao et al. 2019). Therefore, we analyzed whether the amino acids mutation of qGL3 affects its interaction with OsAK3. Our results showed that both qGL3^9311^ and qGL3^N411^ interact with OsAK3 in yeast cells (Fig. 1a).

Glutathione S-transferase (GST) pull down assay was used to study the in vitro interaction between OsAK3 and qGL3. OsAK3 and qGL3 were separately fused with GST- or His- tag to express soluble recombinant proteins in *Escherichia coli*. The western blot showed that GST-OsAK3 strongly bound to His-qGL3 protein (Fig. 1b).

The bimolecular fluorescence complementation (BiFC) was employed for further confirming the in vivo interaction between OsAK3 and qGL3. OsAK3 was fused to the C-terminal fragment of yellow fluorescent protein (YFP) to construct OsAK3-YC, and qGL3 was fused to the N-terminal fragment of YFP to construct qGL3^9311^-YN or qGL3^N411^-YN. OsAK3-YC and qGL3^9311^-YN/qGL3^N411^-YN were co-expressed in *Nicotiana benthamiana* leaves, and the strong YFP fluorescence was observed in the cytoplasm. The empty vector YN or YC co-expressed with OsAK3-YC or qGL3^9311^-YN/qGL3^N411^-YN was used as negative controls (Fig. 1c). These experiments demonstrated that OsAK3 physically interacts with qGL3 in vivo and in vitro.

### Gene Expression, Subcellular Localization and Kinase Activity of OsAK3

**Fig. 2:**
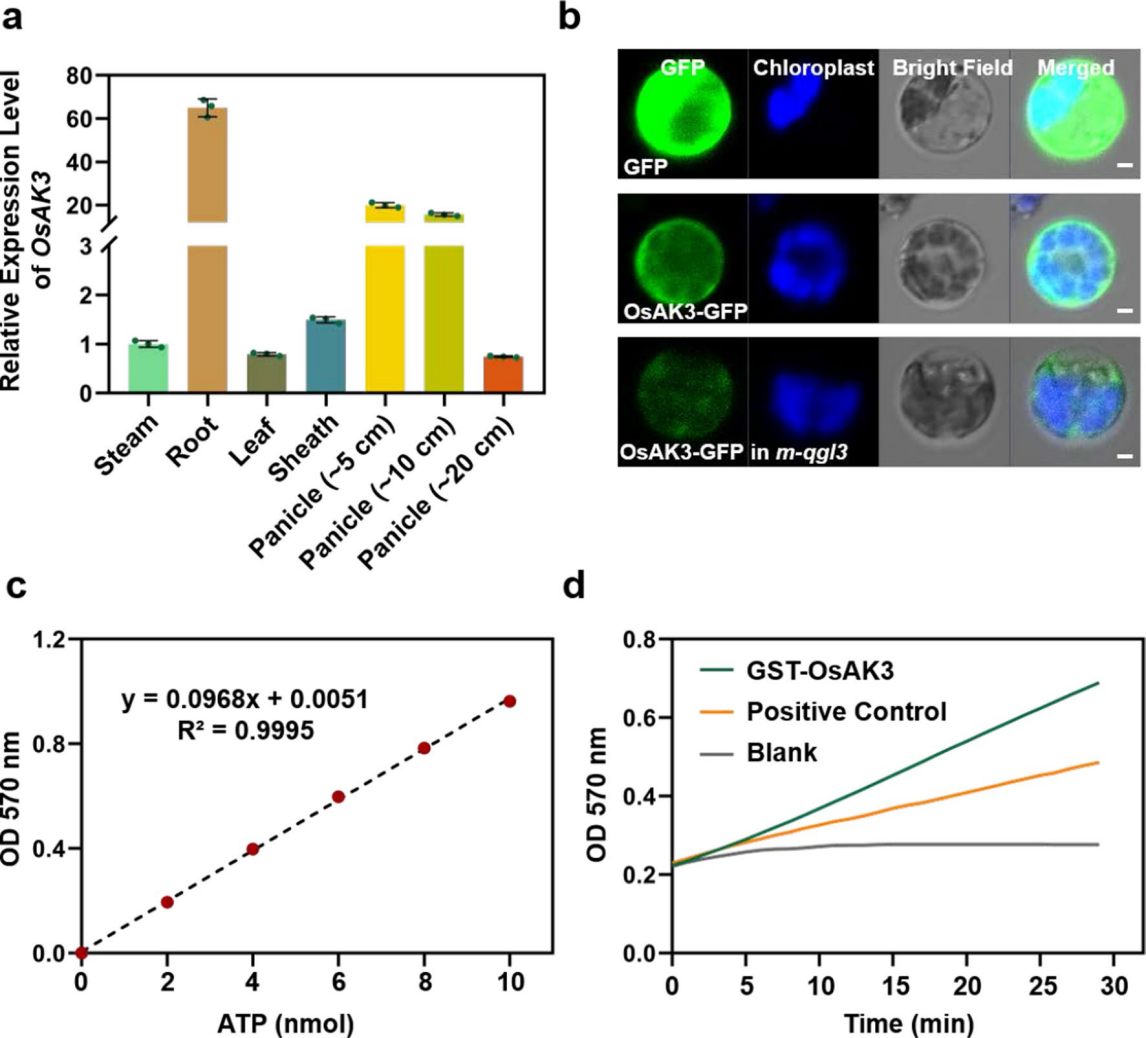
OsAK3 is a functional adenylate kinase localized in cytoplasm. **a** RT-qPCR analysis of *OsAK3* expression in various rice tissues. **b** Subcellular localization of OsAK3 in rice protoplast. *m-qgl3* mutant was used to observe the effect of the absence of qGL3 on OsAK3 localization (scale bar, 2 μm). DJ was used as a wild type. **c** ATP Standard Curve. **d** Colorimetric quantitation of Adenylate Kinase Activity in GST-OsAK3 (0.5 ng) and Positive Control (5 µl)

The quantitative real-time PCR (RT-qPCR) was performed to characterize the expression patterns of *OsAK3* in rice. The results revealed that *OsAK3* gene shows highest expression level in the seedling roots and young panicles (Fig. 2a). To investigate the subcellular localization of OsAK3, we fused OsAK3 to the N-terminus of the green fluorescent protein (GFP). GFP and OsAK3-GFP were transiently expressed in rice protoplasts using PEG-mediated transformation, respectively. OsAK3-GFP signals was observed throughout the cytoplasm (Fig. 2b).

Adenylate kinase activity assay was performed to kinetically measure adenylate kinase activity by detecting adenosine triphosphate (ATP) level, which generated from adenosine diphosphate (ADP). As shown in Fig. 2c, d, OsAK3 has high-level adenylate kinase activity in vitro. According to the ATP production of 14–28 min, the catalytic activity of OsAK3 was calculated to be 341.94 mU/ug. We also examined OsAK3 activity in the presence of qGL3 in vitro. When qGL3 was added, the reduction level of OsAK3 catalytic activity was 19.63 mU/ug. Based on this analysis, we think that qGL3 likely has no significant effect on OsAK3 activity in vitro (Additional file 2: Fig. S1).

### The Homologue OsAK4 also Interacts with qGL3

*OsAK3* encodes an adenylate kinase and has 73.7% homologous with *OsAK4* (Os11g0312220) in nucleotide sequences and 90.8% homologous with *OsAK4* in amino acid sequences (Kawai et al. 1992). As previously stated, there are nine adenylate kinase isoenzymes in human body, which are mainly divided into three categories according to their distribution location (Ionescu 2019). *Arabidopsis thaliana AAK6* has been reported to have the highest homology to human *AK6*, both localized in the nucleus (Slovak et al. 2020). *OsAK1* and *Arabidopsis thaliana AMK2* present closely homologous, both of them are located in chloroplasts and related to photosynthesis (Wei et al. 2017). These results are consistent with our phylogenetic analysis (Additional file 2: Fig. S2a). *Arabidopsis thaliana AMK3* and *AMK4* are the closest homologs to human *AK2*, which is highly expressed in the mitochondrial intermembrane space. However, *AMK3* and *AMK4* were predicted to have no signal target mitochondrial or chloroplast transit peptide (Lange et al. 2008). The phylogenetic analysis showed that *OsAK3* and *OsAK4* belong to the same branch with *Arabidopsis thaliana AMK3* and *AMK4* (Additional file 2: Fig. S2a). Therefore, we also verified whether OsAK4 interacts with qGL3. Y2H analysis showed that OsAK4 also interacts with qGL3 (Additional file 2: Fig. S2b). Similarly, BiFC assay demonstrated that OsAK4 interacts with qGL3 in cytoplasm (Additional file 2: Fig. S2c). These results suggest that adenylate kinases interacting with qGL3 may function redundantly to affect rice growth and development.

### Mutation of *OsAK3* Causes Small Grains and Dwarfism

**Fig. 3:**
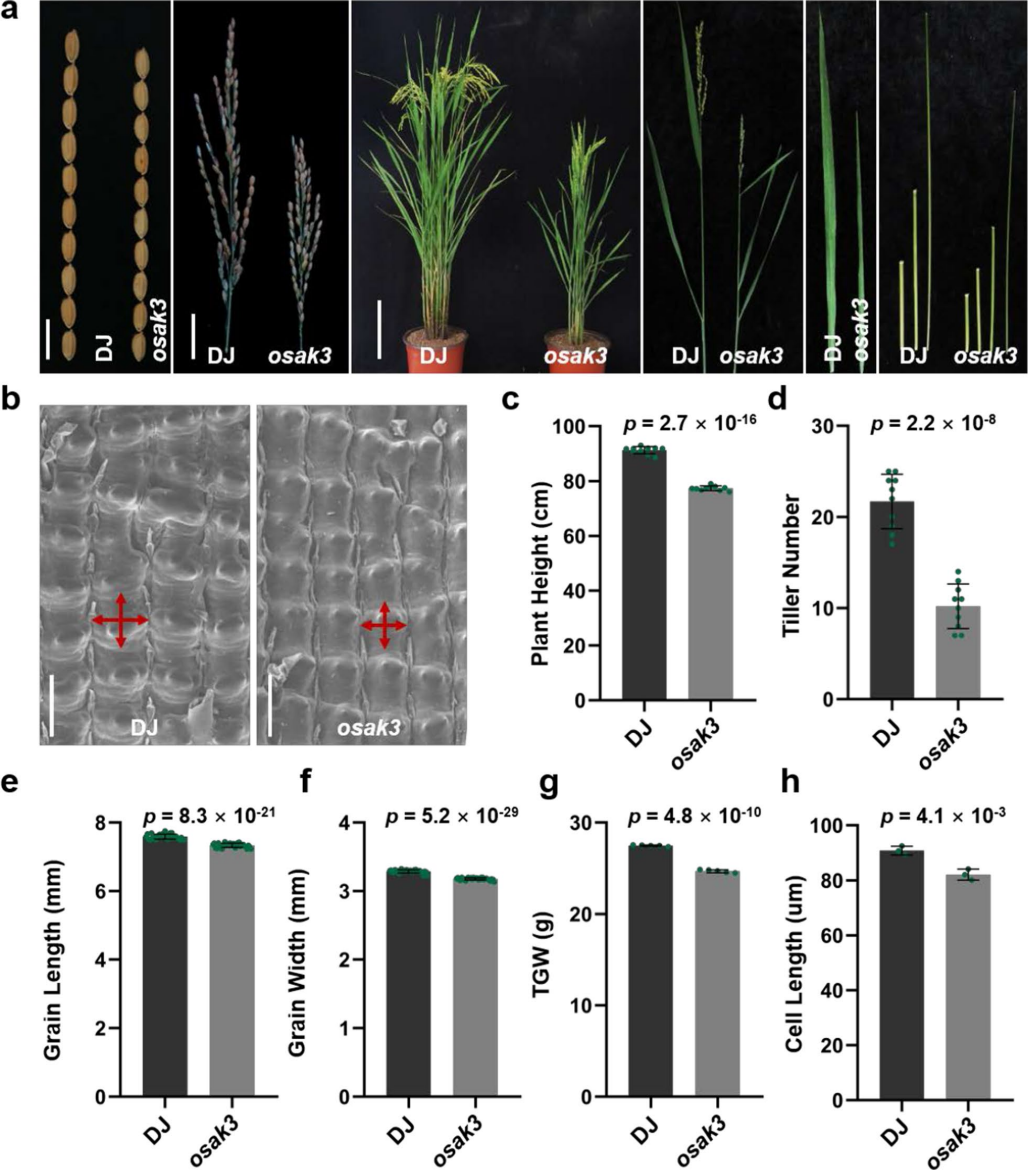
Morphological characteristics of *osak3* mutant. **a** Plant phenotype of DJ and *osak3* at the mature stage. From left to right are grain size (scale bar, 1 cm), panicles (scale bar, 3 cm), plant profile (scale bar, 20 cm), morphology of main axis, flag leaf, internodes. **b** Scanning electron microscopic observation of the spikelet glume cells of DJ and *osak3*. Scale bars, 100 μm. **c, d** Statistical data of plant height (**c**) and tiller number (**d**). Data are means ± SD (*n* = 10). *P* value compared with the wild type by student’s *t* test. **e, f** Statistical data of grain length (**e**) and grain width (**f**). Data are means ± SD (*n* = 30). *P* value compared with the wild type by student’s *t* test. **g** Statistical data of 1000-grain weight. Data are means ± SD (*n* = 5). *P* value compared with the wild type by student’s *t* test. **h** Statistical data of spikelet glume cell length. Data are means ± SD (*n* = 3). *P* value compared with the wild type by student’s *t* test

To explore whether OsAK3 is involved in grain length and BR signaling, we identified a T-DNA insertion mutant *osak3* (Os12g0236400, PFG_3A-01370.L) with a smaller grain size, shorter plant height, and reduced tillers (Fig. 3a, c, d). Compared with wild-type Dongjin (DJ), the average of grain length, width and 1000-grain weight in the *osak3* mutant were significantly decreased (Fig. 3a, e–g). We confirmed the insertion site and the reverse transcription semi-quantitative PCR (RT-sqPCR) identification indicated that the *OsAK3* expression was affected (Additional file 2: Fig. S3a–c). To confirm the effect of the *OsAK3* mutation on rice phenotype, we obtained another line of the T-DNA mutant, *osak3-r* (PFG_3A-01370.R, Additional file 2: Fig. S3d–f) from the rice mutant library. The *osak3-r* also showed a dwarfism phenotype with reduced grain length and width and decreased 1000-grain weight (Additional file 2: Fig S4a, c–g). Since both the size and number of spikelet glume cells affect the final size of the seed (Li et al. 2018), we used scanning electron microscopy (SEM) to examine the glume cells of *osak3*, *osak3-r* and DJ. We found that the average cell length and width of outer glume in *osak3* and *osak3-r* mutants was significantly decreased compared to DJ, and the cell number was remarkably increased (Fig. 3b, h; Additional file 2: Fig. S3g, h, S4b, h–j). These results indicate that *OsAK3* modulates rice grain size by controlling cell expansion.

### Altered BL Sensitivity in *osak3* Mutants

**Fig. 4:**
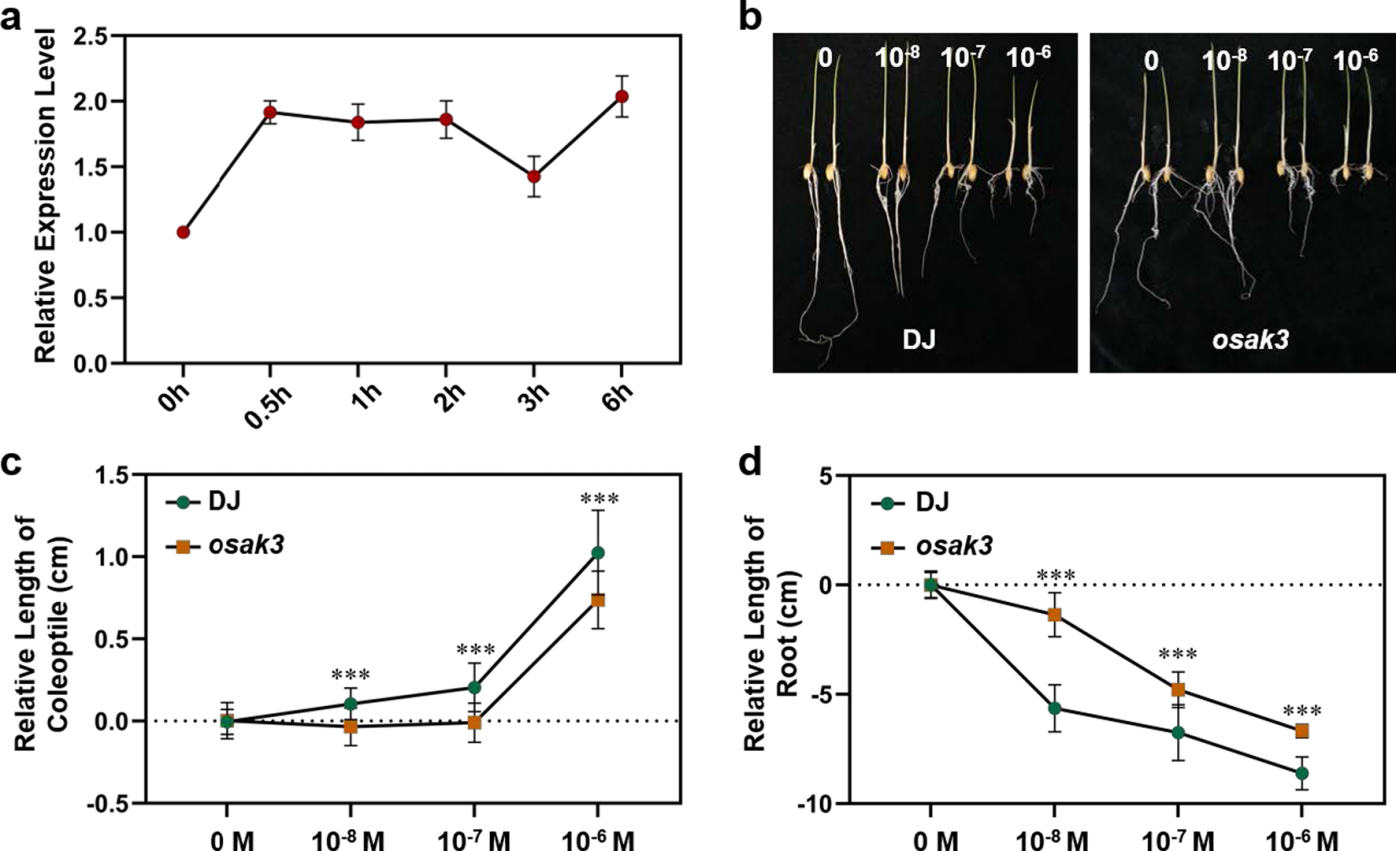
*OsAK3* is involved in rice brassinosteroid signaling. **a** RT-qPCR analysis of *OsAK3* expression under 10^–6^ M BL treatment. Data are means ± SD (*n* = 3). **b** Coleoptile elongation analysis and root inhibition analysis of DJ and *osak3* mutant in response to 0 M, 10^–8^ M,10^–7^ M and 10^–6^ M BL. **c** Statistical data of coleoptile elongation analysis in DJ and *osak3* mutant. The data was analyzed with the relative coleoptile length between BL treatment and mock. Data are means ± SD (*n* = 20). Statistical analyses were performed by student’s *t* test. ***P value < 0.001. **d** Statistical data of root inhibition analysis in DJ and *osak3* mutant. The data was analyzed with the relative root length between BL treatment and mock. Data are means ± SD (*n* = 20). Statistical analyses were performed by student’s *t* test. ***P value < 0.001

Given the presence of BR-deficient phenotypes such as dwarfism, reduced grain length, shorter internodes and increased number of internodes (Fig. 3a; Additional file 2: Fig. S4a), we hypothesized that *OsAK3* is involved in the rice BR pathway. To explore the effect of BR signaling on *OsAK3* expression, DJ was treated with exogenous BL and sampled in time periods for RT-qPCR analysis. These results showed that BL treatment maintained *OsAK3* expression around a twofold level within 6 h (Fig. 4a), indicating that BL induces *OsAK3* expression. To further investigate the relationship between OsAK3 and BR signaling, we examined the sensitivity of *OsAK3* transgenic plants subjected to BL treatment. The coleoptile elongation assay showed that *osak3* mutant had a markedly shorter coleoptile elongation than DJ (Fig. 4b, c). For root elongation assay, as expected, *osak3* was insensitive to BL (Fig. 4b, d). Although the coleoptile of mutant *osak3-r* did not show the same insensitive phenotype to BL treatment, but its root elongation was significantly suppressed compared with DJ (Additional file 2: Fig S5a–c).

We also performed BL-induced lamina inclination experiments. The lamina joint bending of *osak3* mutant showed no obvious change compared with DJ under mock treatment. After incubation in 10^–6^ M BL for 3 days, the lamina joint bending of DJ reached approximately 86°, while that of *osak3* mutant was only about 53° (Additional file 2: Fig. S6a, b), revealing that the mutation of *OsAK3* showed less sensitivity under BL treatment. We also detected endogenous castasterone (CS) level in *osak3* and DJ, and found that CS content was significantly reduced in the mutant (Additional file 2: Fig. S6c).

### Overexpression of *OsAK3* Increased Grain Length

**Fig. 5:**
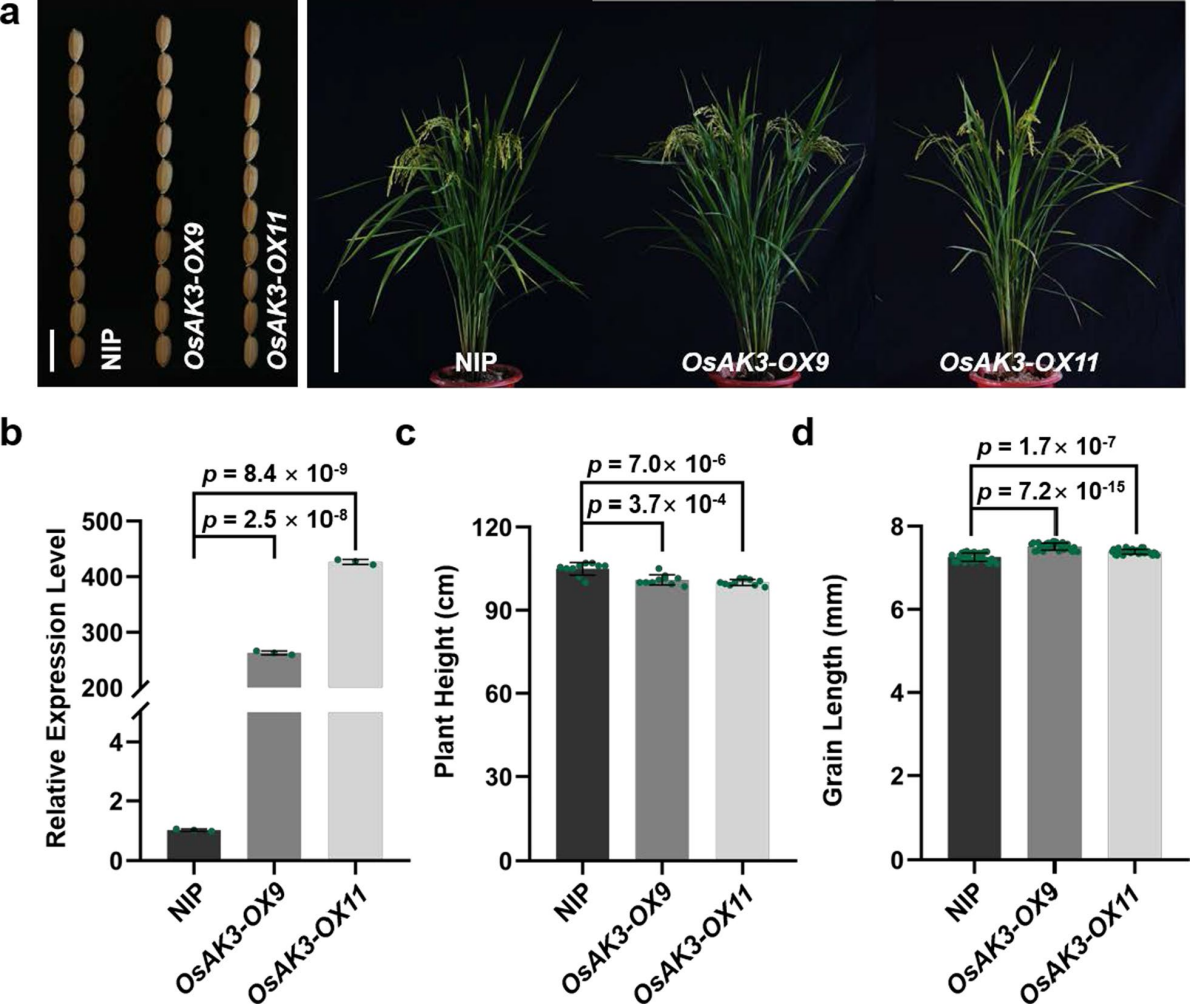
Overexpression of *OsAK3* increased grain length. **a** Plant phenotype of NIP, *OsAK3-OX9* and *OsAK3-OX11* at the mature stage. From left to right are grain size (scale bar, 1 cm) and plant profile (scale bar, 20 cm). **b** RT-qPCR analysis of *OsAK3* expression in two overexpression lines. Data are means ± SD (*n* = 3). *P* value compared with the wild type by student’s *t* test. **c** Statistical data of plant height. Data are means ± SD (*n* = 10). *P* value compared with the wild type by student’s *t* test. **d** Statistical data of grain length. Data are means ± SD (*n* = 30). *P* value compared with the wild type by student’s *t* test

To further validate the function of *OsAK3* in grain development, we constructed an overexpression vector that utilizes the Cauliflower Mosaic Virus 35S promoter to drive the expression of *OsAK3* cDNA. This vector was then transformed into the callus of wild-type Nipponbare (NIP) using *Agrobacterium tumefaciens*-mediated transformation and two transgenic lines were obtained. RT-qPCR analysis showed that the transcript level of *OsAK3* in *OsAK3-OX9* and *OX11* remarkably increased compared to that in NIP (Fig. 5b). Grain length increased significantly and plant height were weakly affected in these two lines compared with NIP (Fig. 5a, c, d). Interestingly, when we performed BL sensitivity analysis on *OsAK3* overexpressing materials, it was found that no significant changes in the coleoptile and root length of *OsAK3-OXs* compared with NIP before and after BL treatment (Additional file 2: Fig. S7a–d).

### Expression of BR Biosynthetic Genes were Affected by OsAK3

**Fig. 6:**
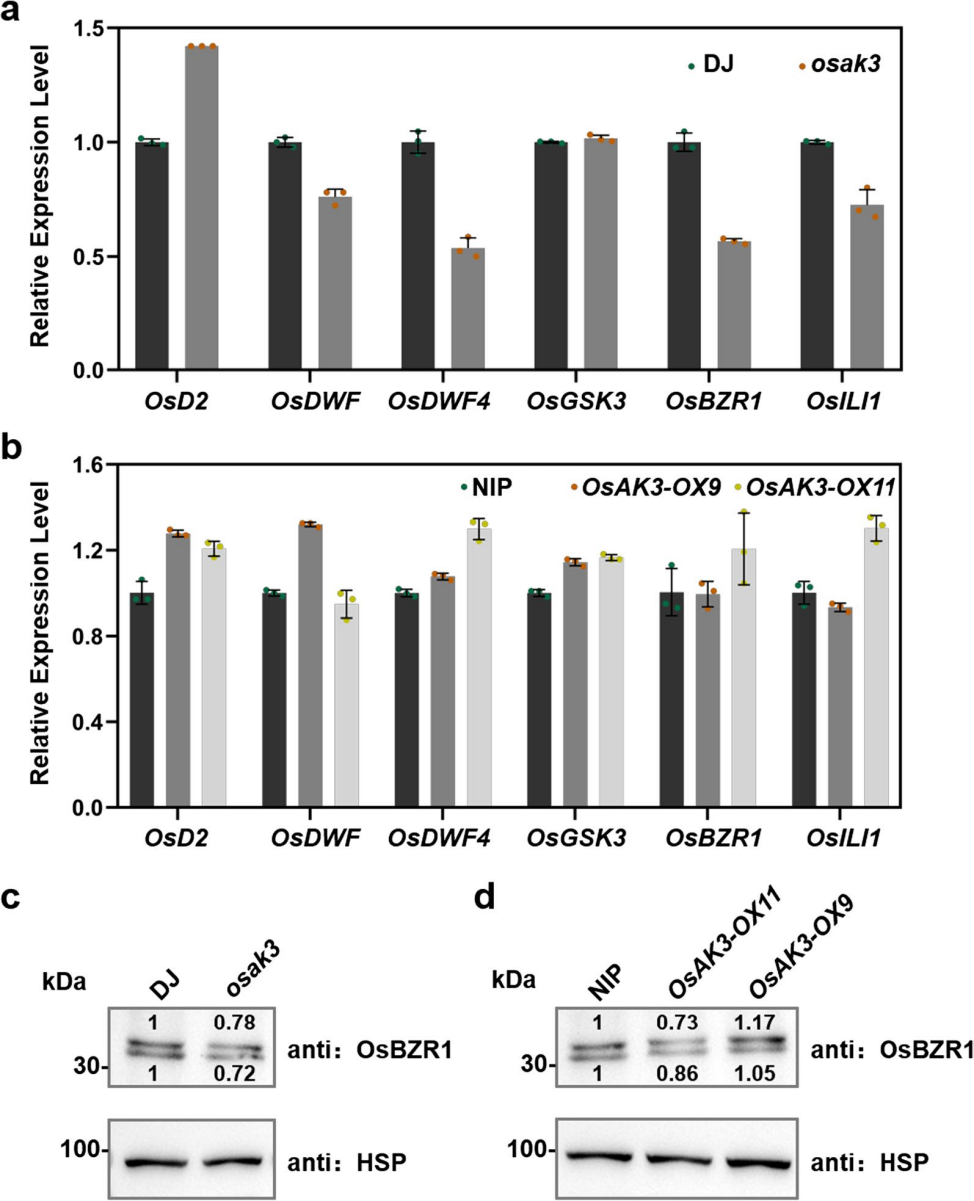
Expression patterns of BR biosynthesis and signaling-related genes were altered in *osak3*.** a** RT-qPCR analysis of *OsD2, OsDWF, OsDWF4, OsGSK3, OsBZR1 *and *OsILI1* expression in DJ and *osak3*. Data are means ± SD (*n* = 3). **b** RT-qPCR analysis of *OsD2, OsDWF, OsDWF4, OsGSK3, OsBZR1 *and *OsILI1* expression in NIP and *OsAK3-OXs* plants. Data are means ± SD (*n* = 3). **c** Western-blot analyses of OsBZR1 protein level in DJ and *osak3*. **d** Western-blot analyses of OsBZR1 protein level in NIP and *OsAK3-OXs* plants

We then examined the expression levels of several BR-related genes in *OsAK3*-related transgenic plants by RT-qPCR (Fig. 6a, b). The expression of BR biosynthetic gene, *OsD2*, was upregulated in *osak3*, but the expression of both *OsDWF* and *OsDWF4* was reduced. The BR signaling related gene *OsBZR1* was also down-regulated in *osak3*. Western blot showed that the protein level of OsBZR1 also decreased in *osak3* (Fig. 6c). OsBZR1 can directly bind to the promoter region of *OsILI1* to activate its expression (Zhang et al. 2009). In *osak3*, the down-regulation of *OsBZR1* expression was followed by a lower *OsILI1* expression (Fig. 6a). The BR synthesis genes were mainly up-expressed in the overexpressed materials (Fig. 6b), and OsBZR1 showed no obvious change in *OsAK3-OX9* plants (Fig. 6b, d).

### Transcriptome Analysis of the *osak3* Mutant by RNA-seq

**Fig. 7:**
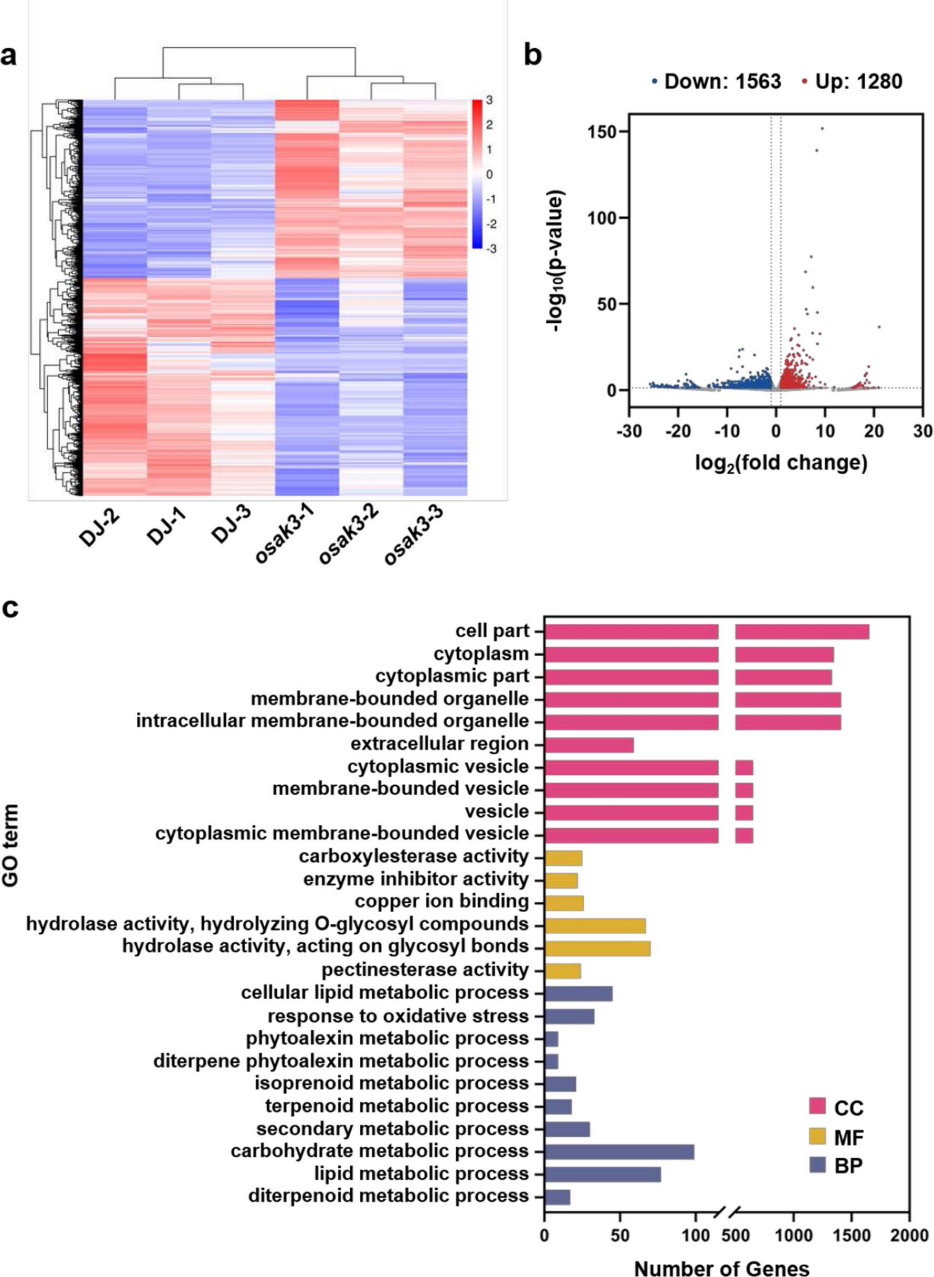
RNA-seq shows that *OsAK3* is involved in multiple metabolic pathways. **a** Hierarchical clustering of the DEGs between DJ and *osak3* mutant plants. Each line with three biological replicates. DEGs were defined by absolute log2 Fold Change > 1 and *P* value < 0.05. Scale bar shows fold changes, values are normalized by z-score scheme, red and blue color indicate up- and down-regulated, respectively. **b** Volcano map of DEGs between DJ and *osak3*. **c** Significantly enriched GO terms of DEGs between DJ and *osak3*. GO terms were sorted based on FDR-adjusted *P* value < 0.05

To further investigate how *OsAK3* functions in rice growth and development, we performed RNA-seq analysis using the young panicles (~ 10 cm) of DJ and *osak3* mutant. An average of ~ 43.6 million clean reads per sample was attained from DJ and *osak3* young panicles cDNA libraries. Cluster analysis of all differentially expressed genes (DEGs) is shown in Fig. 7a. A total of 2843 genes were shown to be differentially expressed between the two genotypes (Fold change > 2, *P* value < 0.05), including 1280 upregulated and 1563 downregulated genes (Fig. 7b). We selected 20 genes for RT-qPCR assay to verify the transcriptomic sequencing results. The expression levels of these 20 genes were consistent with the RNA-seq results, indicating the high quality of the transcriptomic data (Additional file 2: Fig. S8a, b; Additional file 3: Table S2). Gene Ontology (GO) enrichment analysis were performed on all DEGs, and the top 10 terms with the highest significance in biological process (BP), molecular function (MF), and cell component (CC) category were shown in the Fig. 7c. In biological process category, diterpenoid metabolic process, lipid metabolic process, and carbohydrate metabolic process had the smallest FDR (Fig. 7c). Enrichment of DEGs in these processes indicate that *OsAK3* may be involved in regulating lipid and starch metabolic, thus affecting rice grain quality. In molecular function category, DEGs are mainly related to hydrolase activity, hydrolyzing O-glycosyl compounds, and hydrolase activity, acting on glycosyl bonds (Fig. 7c). This suggests that *OsAK3* may affect the function of certain proteins by mediating their glycosylation modifications (Gachon et al. 2005). In cell component category, the most significant GO terms were related to vesicles (Fig. 7c).

**Fig. 8:**
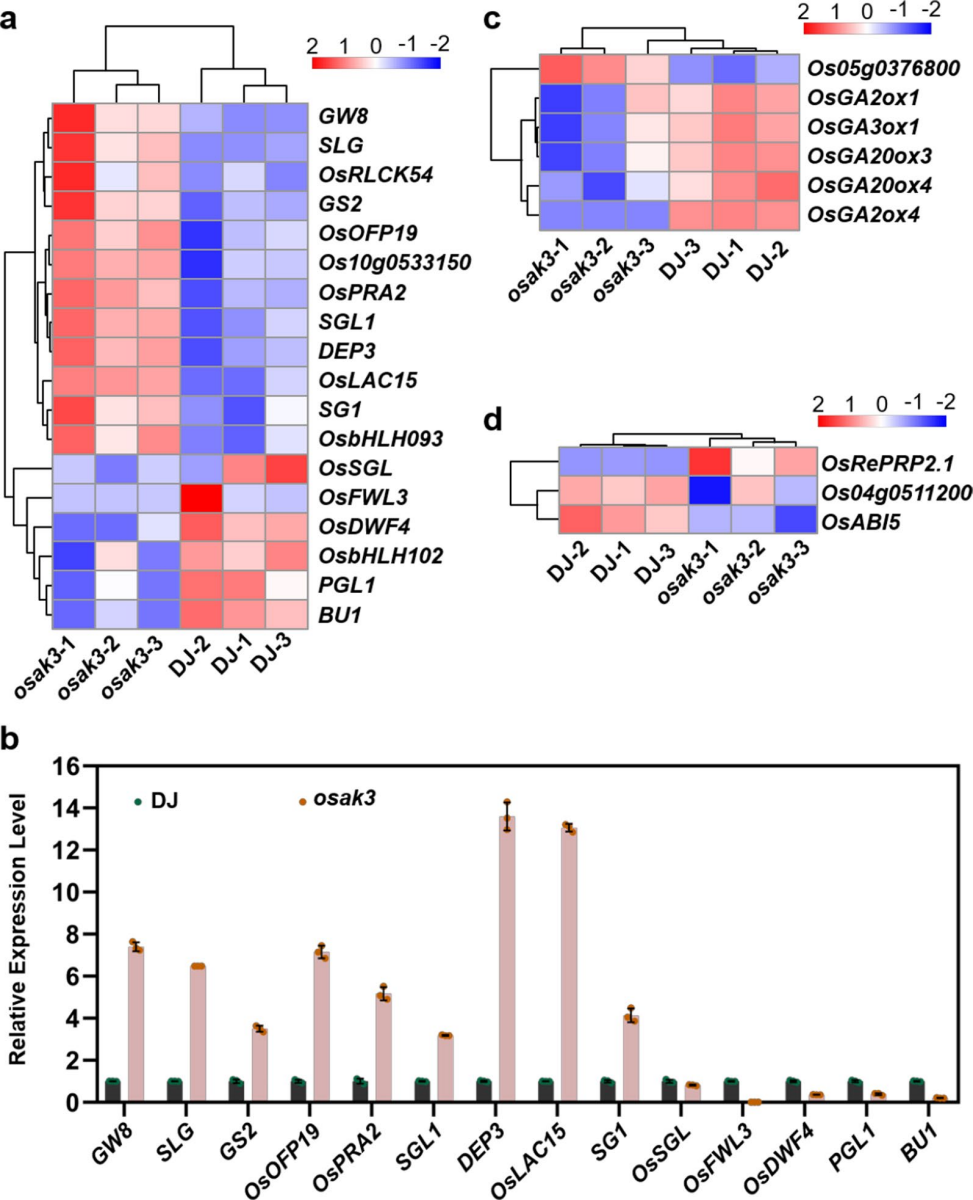
*OsAK3* is involved in multiple phytohormone signaling pathways. **a** Heat map of the grain size- and/or BR-related genes screened from DEGs. Scale bar shows fold changes, values are normalized by z-score scheme, red and blue color indicate up- and down-regulated, respectively. **b** RT-qPCR validation of the grain size- and/or BR-related genes screened from DEGs. Data are means ± SD (*n* = 3). **c, d** Heat map of the DEGs related to gibberellin (**c**) and abscisic acid (**d**). Scale bar shows fold changes, values are normalized by z-score scheme, red and blue color indicate up- and down-regulated, respectively

In particular, the genes involving BR signaling and grain size were analyzed. The rice positive grain size regulators *OsSGL* and *PGL1* were down-regulated in *osak3*, while the expression levels of *GS2*, *GW8* and *SLG* were increased. Similarly, the negative regulator of rice grain size, *SG1*, was up-regulated in *osak3*, but the transcript level of *OsFWL3* was decreased (Fig. 8a, b). This indicates that the mutation of *OsAK3* affected the expression of the grain length and BR response genes. The pathway analysis of DEGs using Mapman software revealed that, in addition to their roles in the BR signaling pathway, DEGs were enriched in other hormone signaling pathways such as auxin, abscisic acid, gibberellin and ethylene (Additional file 2: Fig. S9a–d). We screened genes related to the aforementioned hormone pathways from DEGs for the heat map analysis (Fig. 8c, d and Additional file 2: Fig. S10a–c).

## Discussion

### *OsAK3* Regulates Rice Grain Length and BR Signaling

There are various signaling pathways controlling rice grain size (Zuo and Li 2014; Li et al. 2018). In this study, we obtained two T-DNA insertion mutants *osak3* and *osak3-r* with reduced grain size and decreased plant height, implying the roles of OsAK3 in the regulation of grain length (Fig. 3a, c, e, f; Additional file 2: Fig. S4a, c, e, f). The mutation of *OsAK3* could shorten the length and narrow the width of mutant *osak3* and *osak3-r* glume cells compared with the wild type DJ (Fig. 3b, h; Additional file 2: Fig. S3g, h, S4b, h–j). Therefore, suggesting that *OsAK3* controls rice grain size by regulating cell expansion. Since OsAK3 interacts with qGL3 and qGL3 is a critical modulator in BR signaling pathway, OsAK3 regulates grain length probably through regulating BR signaling.

Therefore, the BL sensitivity assay was performed to study the involvement of OsAK3 in BR signaling. As expected, loss-of-function of *OsAK3* displayed a BR-insensitive phenotype. Above all, lamina joint inclination, coleoptile elongation and root growth inhibition assays indicated that *osak3* was less sensitive to exogenous BL treatment (Fig. 4b–d; Additional file 2: Fig. S6a, b). Interestingly, treatment of exogenous BL had no impact on *OsAK3-OXs* plants (Additional file 2: Fig. S7). We hypothesized that different expression level of *OsAK3* has different effects on BR signaling, since OsAK3 catalyzes a reversible trans-phosphorylation in plant cells. Besides, BL treatment maintains *OsAK3* expression around a twofold higher level within 6 h (Fig. 4a), indicating that BR induces *OsAK3* transcription. Furthermore, the protein level of OsBZR1 decreased in *osak3* mutant compared with wild type. Interestingly, the protein level of OsBZR1 was also down regulated in *OsAK3-OX11*, we speculate that there is a dosage effect due to the higher expression of *OsAK3* in *OsAK3-OX11* compared to *OsAK3-OX9*, which exerts a negative feedback regulation on the protein levels of OsBZR1. Collectively, *OsAK3* is a novel component in rice BR response.

### OsAK3 Physically Interacts with qGL3

Towards an investigation of the molecular mechanism of *OsAK3* in the BR-regulated grain development, the Y2H, GST pull-down and BiFC assays were employed to detect the interaction between OsAK3 and qGL3 (Fig. 1a–c). Although OsAK3 and qGL3 interact with each other, but the alterations in qGL3 phosphatase activity do not affect the interaction, and the absence of qGL3 does not affect the localization of OsAK3 in the cytoplasm (Fig. 2b). qGL3 acts as a protein phosphatase that dephosphorylates OsGSK3 to function in regulating rice grain length. In addition, it has been reported that human AK2 can directly activate the phosphatase activity of DUS26 independently of its AK activity (Kim et al. 2014). Therefore, we also performed dephosphorylation analysis of qGL3 on OsAK3 and the effect of OsAK3 in the dephosphorylation of qGL3 on OsGSK3, unfortunately, we did not obtain a definite result. We propose that OsAK3 interacting with qGL3 affects the interaction intensity between qGL3 and the other qGL3-interacting proteins, such as Cyclin-T1;3 for cell proliferation (Qi et al. 2012). Due to the high sequence similarity between OsAK3 and the homologous OsAK4, we also identified the interaction between OsAK4 and qGL3. The molecular mechanism of the adenylate kinases OsAK3/OsAK4 and qGL3 interactions still remain unclear.

### The Functions of *OsAK3* are Diversified

To explore the role of *OsAK3* in regulating rice growth and development, we performed RNA-seq analysis of *osak3* mutant and DJ. In view of the significant enrichment of DEGs in vesicle components, we also screened some genes related to flower organ identity and development (Paul et al. 2016). We found that the expression of *OsFOR1*, *OsMADS32*, *OsGRF6* and other genes were altered (Additional file 3: Table S3). Mapman analysis suggested that *OsAK3* was involved in biotic and abiotic stress response (Additional file 2: Fig. S9c–e). Previous study showed that salt and submergence stress stimulates adenylate kinase activity (Samarajeewa et al. 1995). DEGs associated with stress response were listed in Additional file 3: Table S4. Based on these results, the potential molecular mechanism of *OsAK3* involved in the stress response is also worth exploring.

OsAK3, as an adenylate kinase, catalyzes a reversible transphosphorylation reaction that converts ADP to ATP and AMP. Adenylate energy charge (AEC) ratio affects cellular energy status, which in turn alters energy-related metabolic processes. GO enrichment showed that a large number of DEGs were related to lipid metabolism and carbohydrate metabolism (Fig. 7c; Additional file 2: Fig. S9a). Thus, *OsAK3* may affect rice quality by regulating lipid and starch content. Terpenoids metabolic pathways were also significantly enriched in GO enrichment (Fig. 7c). Rice diterpenoids play an important role in phytohormone and phytoalexin, such as GA hormone (Wang et al. 2018). We found that GA synthesis genes (*OsGA3ox1*, *OsGA20ox3* and *OxGA20ox4*) were down-regulated along with down-regulated expression of GA inactivation genes (*OsGA2ox1* and *OsGA2ox4*), which may lead to an inhibition on GA biosynthesis (Fig. 8c). Among the secondary metabolic pathways, we found a laccase like protein *OsLAC15* associating with BR response, and previous studies reported that *OsLAC15* overexpressing plants were insensitive to 24-epibrassinolide treatment (Zhang et al. 2013). Heat map and RT-qPCR analysis for the expression of *OsLAC15* and other laccase-related genes revealed that all these genes were up-regulated in the *osak3* mutant. Thus, *OsAK3* may respond to BR signaling by regulating the expression of *OsLACs* (Additional file 2: Fig. S10d, e). Taken together, we propose a working model for the response of OsAK3 to BR signals in rice (Additional file 2: Fig. S11).

## Conclusions

In this study, we found that *OsAK3* regulates grain size through controlling spikelet glume cells expansion. OsAK3 participates in BR signaling response and interacts with qGL3. RNA-seq analysis revealed potential functions of *OsAK3* in rice floral organ identity, stress responses and various biological processes. These results give insight into the function of adenylate kinase *OsAK3* and extend our understanding of BR signaling pathway.

## Materials and Methods

### Plant Materials and Growth Conditions

The T-DNA insertion mutant *osak3* (PFG_3A-01370.L) and *osak3-r* (PFG_3A-01370.R) was obtained from the Korean rice mutant library (https://signal.salk.edu). The *japonica* (*Oryza sativa*) cultivar Dongjin (DJ) was the wild-type control. To generate *OsAK3*-overexpression transgenic plants, the full-length coding sequence of *OsAK3* was linked to the pCAMBIA-1300s plasmid to construct the 35S: *OsAK3* vector. Then the vector was transformed into *Agrobacterium tumefaciens* (EHA105) and introduced into *japonica* (*Oryza sativa*) cultivar Nipponbare (NIP) by *Agrobacterium*-mediated transformation method. Rice plants were cultivated in the field under natural long days in Nanjing, China. The agronomic traits of homozygous rice plants were investigated before harvest.

### Total RNA Isolation and RT-qPCR Analysis

Total RNA was extracted with a High Purity Total RNA Rapid Extraction Kit (Tiangen, Beijing, China) according to the manufacturer’s instructions. First-strand cDNA was synthesized using HiScript® II Q RT SuperMix for qPCR (+ gDNA wiper) Kit (Vazyme, Nanjing, China). Real-time quantitative PCR (RT-qPCR) was performed using AceQ® qPCR SYBR Green Master Mix Kit (Vazyme, Nanjing, China) and Roche 480 Real-Time PCR System following the manufacturer’s instructions. The rice *OsActin* gene (LOC_Os03g50885) was used as an internal control and for the normalization in the analysis. The relative gene expression level was calculated using the 2^−ΔΔCt^ method as previously reported (Gao et al. 2019). The data were presented as the mean ± _SD_ of three replicates. The primers for RT-sqPCR and RT-qPCR are listed in Additional file 3: Table S1.

### Yeast Two-Hybrid Assay

The full-length coding sequence of *OsAK3* and *OsAK4* was cloned into pGADT7, respectively to form the prey construct AD-OsAK3 and AD-OsAK4. The respective combinations of vectors with bait construct BD-qGL3 were co-transformed into the yeast strain AH109 according to the manufacturer’s instruction (Clontech, USA). The transformants were grown on synthetic defined medium (SD)/-Trp-Leu at 30 °C for 3 days and the interaction was confirmed by the colony growth in SD/-Trp-Leu-His-Ade with 5-Bromo-4-Chloro-3-Indolyl-α-D-Galactoside (X-α-gal). The PCR primers used for yeast two-hybrid assay are listed in Additional file 3: Table S1.

### Bimolecular Fluorescence Complementation (BiFC) Assay

For BiFC assays, *OsAK3* and *OsAK4* were cloned into the p2YC vector and *qGL3* was cloned into p2YN vector, resulting in OsAK3-YC, OsAK4-YC, qGL3^9311^-YN and qGL3^N411^-YN. These recombinant plasmids and empty vectors were transformed into *Agrobacterium tumefaciens* strain EHA105. The corresponding *Agrobacterium* cells combination were injected into young leaves of *Nicotiana benthamiana*. The fluorescence was observed under a Zeiss LSM780 confocal microscope after growth for 36–48 h in darkness. The PCR primers used for BiFC assay are listed in Additional file 3: Table S1.

### In Vitro GST Pull-Down Assay

To verify the in vitro interaction between OsAK3 and qGL3, the full-length coding sequence of *OsAK3* was cloned into the pGEX-2T vector and transformed into the *Escherichia coli* strain BL21 (DE3) to express the GST-OsAK3 fusion proteins. The full-length coding sequence of *qGL3* was cloned into the pET-30a vector and transformed into the *Escherichia coli* strain BL21 (DE3) to express the His-qGL3 fusion proteins. Fusion proteins GST-OsAK3 and His-qGL3 were induced with 0.5 mM isopropyl-b-D-thiogalactopyranoside (IPTG) at 18 °C for 12 h. For GST pull-down assay, bacterial lysates containing GST-OsAK3 or GST were mixed with lysates containing His-qGL3. Subsequently, GST Bind Resin (Novagen) was added to the miscible liquids and incubated with the fusion proteins for 4 h. Beads were washed three times and then boiled in 1 × SDS loading buffer for 10 min. Finally, the mixture was separated by 10% SDS-PAGE. The GST antibody (Cell Signaling Technology) and His antibody (Cell Signaling Technology) were used to detect the proteins by Western blot analysis. The PCR primers used for GST pull-down assay are listed in Additional file 3: Table S1.

### Exogenous BL Treatment

For coleoptile elongation analysis, rice seeds were grown on 0.3% agar medium with different concentrations BL after germination. Coleoptile lengths were measured after 5 days grown in darkness. For lamina inclination assays, segments of the second leaf blade, lamina joint and 1 cm of leaf sheath were cut off from 1-week-old dark grown rice and inserted vertically into the 0.3% agar medium with different concentrations BL. The angles of lamina joint bending were measured after 72 h grown in dark. For root inhibition analysis, rice seeds were sowed and grown in the solution culture with different concentrations BL after germination. Root lengths were measured after 5 days grown in darkness. For *OsAK3* transcript measurement, 1-week-old DJ seedlings were treated with 10^–6^ M BL and sampled at 0, 0.5, 1, 2, 3 and 6 h.

### Subcellular Localization

To determine the subcellular localization of OsAK3, the full-length OsAK3 coding sequence was fused with green fluorescent protein (GFP) in the pAN580 vector to produce the OsAK3-GFP fusion protein in plants. Rice protoplasts were isolated from 2-week-old rice seedlings and transfected with 10 µg plasmid DNA by PEG-mediated transformation methods. The GFP signal was visualized using a confocal laser scanning microscope (LSM780, Zeiss, Germany) after incubation at 26 °C for 12 h. The PCR primers used for transient expression assay are listed in Additional file 3: Table S1.

### Scanning Electron Microscopy Observation of Spikelet Hull

Mature rice seeds of WT and transgenic lines were collected and were fixed in FAA solution, dehydrated in series concentrations of ethanol, and critical point-dried in a vacuum freeze drier. Subsequently, samples were mounted, coated with gold, and finally observed under a scanning electron microscopy (Hitachi, Japan). The cell size of each sample was measured using ImageJ software.

### RNA-Seq Analysis

Young panicles of WT and *osak3* mutant were sampled for RNA-seq analysis with three biological replicates. The extraction and examination of total RNA, library construction and Illumina sequencing were done by Personal Biotech (Shanghai, China) using the Illumina novaseq pe150. The clean reads were aligned with reference sequences of rice in IRGSP-1.0 (http://rapdb.dna.affrc.go.jp/download/irgsp1.html). Differentially expressed genes (DEGs) were defined by absolute log_2_ Fold Change > 1 and *P* value < 0.05. The DEGs were classified according to Gene Ontology (GO) annotation using AgriGO (http://bioinfo.cau.edu.cn/agriGO). The selected differential expressed genes were blasted using the RGAP database (http://rice.plantbiology.msu.edu/) and the NCBI database (https://www.ncbi.nlm.nih.gov/).

### Phylogenetic Analysis

The sequences of rice, Arabidopsis and human adenylate kinases were downloaded from NCBI (https://www.ncbi.nlm.nih.gov/). Multiple sequence alignments of these homologs were performed using Muscle (MEGA6) and the phylogenetic tree was constructed using the Neighbor-Joining method (MEGA6). Bootstrap values were obtained by 1,000 bootstrap replicates. The amino acid sequences used for phylogenetic analysis are listed in Additional file 1: Supplemental Data Set 1.

## Supplementary Information

**Additional file 1: Supplemental Data Set 1. **Adenylate kinase sequences used in phylogenetic tree analysis.

**Additional file 2: Fig. S1. **Colorimetric quantitation of OsAK3 activity in the presence of qGL3 in vitro. **Fig. S2.** OsAK4 has the highest homology with OsAK3 and interacts with qGL3. **Fig. S3.** Genotyping analysis of *osak3* and *osak3-r* mutant. **Fig. S4.** Morphological characteristics of *osak3-r* mutant. **Fig. S5.** Effects of BL treatment in *osak3-r*. **Fig. S6.** Effects of BL treatment in *osak3*. **Fig. S7.** Effects of BL treatment in *OsAK3-OXs* plants. **Fig. S8.** RT-qPCR verification of DEGs selected from RNA-seq. **Fig. S9.** Overview of the DEGs between DJ and *osak3* mutant. **Fig. S10.** *OsAK3* is involved in multiple phytohormone signaling pathways and stress responses. **Fig. S11.** A working model for the functions of OsAK3 in BR signaling and plant growth and development.

**Additional file 3: Table S1.** Primers used in this study. **Table S2.** List of DEGs used for RT-qPCR validation. **Table S3.** List of DEGs associated with floral organ development detected in DJ and *osak3*. **Table S4.** List of DEGs associated with stress response detected in DJ and *osak3*.

## Data Availability

The datasets supporting the conclusions of this article are included within the article and its additional files.

## Abbreviations

BRs: Brassinosteroids
BL: Brassinolide
CS: Castasterone
GIP: qGL3-interacting protein
TF: Transcription factor
AK: Adenylate kinase
ATP: Adenosine triphosphate
ADP: Adenosine diphosphate
DJ: Dongjin
NIP: Nipponbare
PADK1: PLASTIDIAL ADENYLATE KINASE 1
AMK2: ADENOSINE MONOPHOSPHATE KINASE 2
Y2H: Yeast two-hybrid
RT-sqPCR: Reverse transcription semi-quantitative PCR
RT-qPCR: Quantitative real-time PCR
GFP: Green fluorescent protein
GST: Glutathione S-transferase
BiFC: Bimolecular fluorescence complementation
YFP: Yellow fluorescent protein
SEM: Scanning electron microscopy
DEG: Differentially expressed gene
GO: Gene Ontology
BP: Biological process
MF: Molecular function
CC: Cell component
AEC: Adenylate energy charge
