# Supplementary material for: An Adenylate Kinase OsAK3 Involves Brassinosteroid Signaling and Grain Length in Rice (Oryza sativa L.)
Source: Rice (N Y). 2021 Dec 28;14:105. doi: 10.1186/s12284-021-00546-0 (PMC8714616; doi:10.1186/s12284-021-00546-0)
Supplement: Supplementary file 1 — Additional file 1: Supplemental Data Set 1. Adenylate kinase sequences used in phylogenetic tree analysis. [file 12284_2021_546_MOESM1_ESM.docx]

**Supplemental Data Set 1. Adenylate kinase sequences used in phylogenetic tree analysis.**

>Adenylate kinase 1 (Homo sapiens)

MEEKLKKTNIIFVVGGPGSGKGTQCEKIVQKYGYTHLSTGDLLRSEVSSGSARGKKLSEIMEKGQLVPLETVLDMLRDAMVAKVNTSKGFLIDGYPREVQQGEEFERRIGQPTLLLYVDAGPETMTQRLLKRGETSGRVDDNEETIKKRLETYYKATEPVIAFYEKRGIVRKVNAEGSVDSVFSQVCTHLDALK

>Adenylate kinase 2 (Homo sapiens)

MAPSVPAAEPEYPKGIRAVLLGPPGAGKGTQAPRLAENFCVCHLATGDMLRAMVASGSELGKKLKATMDAGKLVSDEMVVELIEKNLETPLCKNGFLLDGFPRTVRQAEMLDDLMEKRKEKLDSVIEFSIPDSLLIRRITGRLIHPKSGRSYHEEFNPPKEPMKDDITGEPLIRRSDDNEKALKIRLQAYHTQTTPLIEYYRKRGIHSAIDASQTPDVVFASILAAFSKATCKDLVMFI

>Adenylate kinase 3 (Homo sapiens)

MGASARLLRAVIMGAPGSGKGTVSSRITTHFELKHLSSGDLLRDNMLRGTEIGVLAKAFIDQGKLIPDDVMTRLALHELKNLTQYSWLLDGFPRTLPQAEALDRAYQIDTVINLNVPFEVIKQRLTARWIHPASGRVYNIEFNPPKTVGIDDLTGEPLIQREDDKPETVIKRLKAYEDQTKPVLEYYQKKGVLETFSGTETNKIWPYVYAFLQTKVPQRSQKASVTP

>Adenylate kinase 4 (Homo sapiens)

MASKLLRAVILGPPGSGKGTVCQRIAQNFGLQHLSSGHFLRENIKASTEVGEMAKQYIEKSLLVPDHVITRLMMSELENRRGQHWLLDGFPRTLGQAEALDKICEVDLVISLNIPFETLKDRLSRRWIHPPSGRVYNLDFNPPHVHGIDDVTGEPLVQQEDDKPEAVAARLRQYKDVAKPVIELYKSRGVLHQFSGTETNKIWPYVYTLFSNKITPIQSKEAY

>Adenylate kinase 5 (Homo sapiens)

MCSKPEDPVEYLESCLQKVKELGGCDKVKWDTFVSQEKKTLPPLNGGQSRRSFLRNVMPENSNFPYRRYDRLPPIHQFSIESDTDLSETAELIEEYEVFDPTRPRPKIILVIGGPGSGKGTQSLKIAERYGFQYISVGELLRKKIHSTSSNRKWSPIAKIITTGELAPQETTITEIKQKLMQIPDEEGIVIDGFPRDVAQALSFEDQICTPDLVVFLACANQRLKERLLKRAEQQGRPDDNVKATQRRLMNFKQNAAPLVKYFQEKGLIMTFDADRDEDEVFYDISMAVDNKLFPNKEAAAGSSDLDPSMILDTGEIIDTGYDYEDQGDDQLNVFGEDTMGGFMEDLRKCKIIFIIGGTGSGKGTQCEKLVEKYGFTHLSTGELLREELASESERSKLIRDIMERGDLVPSGIVLELLKEAMVASLGDTRGFLIDGYPREVKQGEEFGRRIGDPQLVICMDCSADTMTNRLLQRSRSSLPVDDTTKTIAKRLEAYYRASIPVIAYYETKTQLHKINAEGTPEDVFLQLCTAIDSIIF

>Adenylate kinase 6 (Homo sapiens)

MCSKPEDPVEYLESCLQKVKELGGCDKVKWDTFVSQEKKTLPPLNGGQSRRSFLRNVMPENSNFPYRRYDRLPPIHQFSIESDTDLSETAELIEEYEVFDPTRPRPKIILVIGGPGSGKGTQSLKIAERYGFQYISVGELLRKKIHSTSSNRKWSPIAKIITTGELAPQETTITEIKQKLMQIPDEEGIVIDGFPRDVAQALSFEDQICTPDLVVFLACANQRLKERLLKRAEQQGRPDDNVKATQRRLMNFKQNAAPLVKYFQEKGLIMTFDADRDEDEVFYDISMAVDNKLFSNKEAAAGSSDLDPSMILDTGETIDTGSDYEDQGDDQLNVFGEDTMGGFMEDLRKCKIIFIIGGPGSGKGTQCEKLVEKYGFTHLSTGELLREELASESERSKLIRDIMERGDLVPSGIVLELLKEAMVASLGDTRGFLIDGYPREVKQGEEFGRRIGDPQLVICMDCSADTMTNRLLQRSRSSLPVDDTTKTIAKRLEAYYRASIPVIAYYETKTQLHKINAEGTPEDVFLQLCTAIDSIF

>AD-004 adenylate kinase 6 (Homo sapiens)

MLLPNILLTGTPGVGKTTLGKELASKSGLKYINVGDLAREEQLYDGYDEEYDCPILDEDRVVDELDNQMREGGVIVDYHGCDFFPERWFHIVFVLRTDTNVLYERLETRGYNEKKLTDNIQCEIFQVLYEEATASYKEEIVHQLPSNKPEELENNVDQILKWIEQWIKDHNS

>Adenylate kinase 7 isoform 1 (Homo sapiens)

MAEEEETAALTEKVIRTQRVFINLLDSYSSGNIGKFLSNCVVGASLEEITEEEEEEDENKSAMLEASSTKVKEGTFQIVGTLSKPDSPRPDFAVETYSAISREDLLMRLLECDVIIYNITESSQQMEEAIWAVSALSEEVSHFEKRKLFILLSTVMTWARSKALDPEDSEVPFTEEDYRRRKSHPNFLDHINAEKMVLKFGKKARKFAAYVVAAGLQYGAEGGMLHTFFKMAWLGEIPALPVFGDGTNVIPTIHVLDLAGVIQNVIDHVPKPHYLVAVDESVHTLEDIVKCISKNTGPGKIQKIPRENAYLTKDLTQDCLDHLLVNLRMEALFVKENFNIRWAAQTGFVENINTILKEYKQSRGLMPIKICILGPPAVGKSSIAKELANYYKLHHIQLKDVISEAIAKLEAIVAPNDVGEGEEEVEEEEEEENVEDAQELLDGIKESMEQNAGQLDDQYIIRFMKEKLKSMPCRNQGYILDGFPKTYDQAKDLFNQEDEEEEDDVRGRMFPFDKLIIPEFVCALDASDEFLKERVINLPESIVAGTHYSQDRFLRALSNYRDINIDDETVFNYFDELEIHPIHIDVGKLEDAQNRLAIKQLIKEIGEPRNYGLTDEEKAEEERKAAEERLAREAAEEAEREHQEAVEMAEKIARWEEWNKRLEEVKREERELLEAQSIPLRNYLMTYVMPTLIQGLNECCNVRPEDPVDFLAEYLFKNNPEAQ

>Adenylate kinase 8 isoform 1 (Homo sapiens)

MDATIAPHRIPPEMPQYGEENHIFELMQNMLEQLLIHQPEDPIPFMIQHLHRDNDNVPRIVILGPPASGKTTIAMWLCKHLNSSLLTLENLILNEFSYTATEARRLYLQRKTVPSALLVQLIQERLAEEDCIKQGWILDGIPETREQALRIQTLGITPRHVIVLSAPDTVLIERNLGKRIDPQTGEIYHTTFDWPPESEIQNRLMVPEDISELETAQKLLEYHRNIVRVIPSYPKILKVISADQPCVDVFYQALTYVQSNHRTNAPFTPRVLLLGPVGSGKSLQAALLAQKYRLVNVCCGQLLKEAVADRTTFGELIQPFFEKEMAVPDSLLMKVLSQRLDQQDCIQKGWVLHGVPRDLDQAHLLNRLGYNPNRVFFLNVPFDSIMERLTLRRIDPVTGERYHLMYKPPPTMEIQARLLQNPKDAEEQVKLKMDLFYRNSADLEQLYGSAITLNGDQDPYTVFEYIESGIINPLPKKIP

>Adenylate kinase 9 isoform 1 (Homo sapiens)

MTSQEKTEEYPFADIFDEDETERNFLLSKPVCFVVFGKPGVGKTTLARYITQAWKCIRVEALPILEEQIAAETESGVMLQSMLISGQSIPDELVIKLMLEKLNSPEVCHFGYIITEIPSLSQDAMTTLQQIELIKNLNLKPDVIINIKCPDYDLCQRISGQRQHNNTGYIYSRDQWDPEVIENHRKKKKEAQKDGKGEEEEEEEEQEEEEAFIAEMQMVAEILHHLVQRPEDYLENVENIVKLYKETILQTLEEVMAEHNPQYLIELNGNKPAEELFMIVMDRLKYLNLKRAAILTKLQGAEEEINDTMENDELFRTLASYKLIAPRYRWQRSKWGRTCPVNLKDGNIYSGLPDYSVSFLGKIYCLSSEEALKPFLLNPRPYLLPPMPGPPCKVFILGPQYSGKTTLCNMLAENYKGKVVDYAQLVQPRFDKARETLVENTIAEATAAAIKVVKEKLLRELQARKQAETALREFQRQYEKMEFGVFPMEATHSSIDEEGYIQGSQRDRGSSLVDTEEAKTKSENVLHDQAAKVDKDDGKETGETFTFKRHSQDASQDVKLYSDTAPTEDLIEEVTADHPEVVTMIEETIKMSQDINFEQPYEKHAEILQEVLGEVMEENKDRFPGAPKYGGWIVDNCPIVKELWMALIKKGIIPDLVIYLSDTENNGKCLFNRIYLQKKSEIDSKILERLLEELQKKKKEEEEARKATEEELRLEEENRRLLELMKVKAKEAEETDNEDEEEIEGDELEVHEEPEASHDTRGSWLPEEFEASEVPETEPEAVSEPIEETTVETEIPKGSKEGLEIEKLSETVVLPEFPEDSYPDVPEMEPFKEKIGSFIILWKQLEATISEAYIKILNLEIADRTPQELLQKVVETMEKPFQYTAWELTGEDYEEETEDYQTEAEVDEELEEEEEEEGEDKMKERKRHLGDTKHFCPVVLKENFILQPGNTEEAAKYREKIYYFSSAEAKEKFLEHPEDYVAHEEPLKAPPLRICLVGPQGSGKTMCGRQLAEKLNIFHIQFEEVLQEKLLLKTEKKVGPEFEEDSENEQAAKQELEELAIQANVKVEEENTKKQLPEVQLTEEEEVIKSSLMENEPLPPEILEVILSEWWLKEPIRSTGFILDGFPRYPEEAQFLGDRGFFPDAAVFIQVDDQDIFDRLLPAQIEKWKLKQKKKLERKKLIKDMKAKIRVDTIAKRRAELILERDKKRRENVVRDDEEISEEELEEDNDDIENILEDEFPKDEEEMSGEEDEEQETDAIERLRGELGEKFEADTHNLQIIQDELERYLIPIISINGARRNHIVQYTLNMKLKPLVENRASIFEKCHPIPAPLAQKMLTFTYKYISSFGYWDPVKLSEGETIKPVENAENPIYPVIHRQYIYFLSSKETKEKFMKNPIKYIRQPKPKPTVPIRIIIVGPPKSGKTTVAKKITSEYGLKHLSIGGALRYVLNNHPETELALMLNWHLHKGMTAPDELAIQALELSLMESVCNTAGVVIDGYPVTKHQMNLLEARSIIPMVIFELSVPSKEIFKRLLLEKENEQRLPYPLHNSAQIVAVNNVKYRKNIGEIRQYYQEQHQNWYVIDGFHSKWWVWNEVIKNVQMVNKYMQTYLERIKAGKAACIDKLCITPQELLSRLGEFEQFCPVSLAESQELFDCSATDSLEFAAEFRGHYYKMSSQEKLNKFLENPELYVPPLAPHPLPSADMIPKRLTLSELKSRFPKCAELQGYCPVTYKDGNQRYEALVPGSINYALEYHNRIYICENKEKLQKFLRSPLKYWEQKLPHKLPPLREPILLTSLPLPGYLEQGIATSLIKAMNAAGCLKPKFPFLSIRRSALLYIALHLKAFNPKGSEYTRKKYKKKMEQFMESCELITYLGAKMTRKYKEPQFRAIDFDHKLKTFLSLRNIDPING

>Adenylate kinase 9 isoform 2 (Homo sapiens)

MTSQEKTEEYPFADIFDEDETERNFLLSKPVCFVVFGKPGVGKTTLARYITQAWKCIRVEALPILEEQIAAETESGVMLQSMLISGQSIPDELVIKLMLEKLNSPEVCHFGYIITEIPSLSQDAMTTLQQIELIKNLNLKPDVIINIKCPDYDLCQRISGQRQHNNTGYIYSRDQWDPEVIENHRKKKKEAQKDGKGEEEEEEEEQEEEEAFIAEMQMVAEILHHLVQRPEDYLENVENIVKLYKETILQTLEEVMAEHNPQYLIELNGNKPAEELFMIVMDRLKYLNLKRAAILTKLQGAEEEINDTMENDELFRTLASYKLIAPRYRWQRSKWGRTCPVNLKDGNIYSGLPDYSVSFLGKIYCLSSEEALKPFLLNPRPYLLPPMPGPPCKVFILGPQYSGKTTLCNMLAENYKGKVTN

>AAK6 (Arabidopsis thaliana)

MARRNRGVTRRERPNLLITGTPGTGKSTTASALAEATNLRYICIGDLVKEKEFYHGWDNELECHFINEDSVIDELDDAMIEGGNIVDYHGCDFFPQRWFDRVVVLRTENSVLYDRLTNRGYSGTKLSNNLQCEMYQVLLEEAHDSYDEEIVTELQSNTIEDISNNVSTLTDWINAWQP

>AMK1(Arabidopsis thaliana)

MARLVRVARSSSLFGFGNRFYSTSAEASHASSPSPFLHGGGASRVAPKDRNVQWVFLGCPGVGKGTYASRLSTLLGVPHIATGDLVREELASSGPLSQKLSEIVNQGKLVSDEIIVDLLSKRLEAGEARGESGFILDGFPRTMRQAEILGDVTDIDLVVNLKLPEEVLVDKCLGRRTCSQCGKGFNVAHINLKGENGRPGISMDPLLPPHQCMSKLVTRADDTEEVVKARLRIYNETSQPLEEYYRTKGKLMEFDLPGGIPESWPRLLEALRLDDYEEKQSVAA

>AMK2(Arabidopsis thaliana)

MTGCVNSISPPPVTLYRHRASPSRSSFSLSGDALHSLYRHRRVSRSPSIIAPKFQIVAAEKSEPLKIMISGAPASGKGTQCELITHKYGLVHISAGDLLRAEIASGSENGRRAKEHMEKGQLVPDEIVVMMVKDRLSQTDSEQKGWLLDGYPRSASQATALKGFGFQPDLFIVLEVPEEILIERVVGRRLDPVTGKIYHLKYSPPETEEIAVRLTQRFDDTEEKAKLRLKTHNQNVSDVLSMYDDITIKIEGNRSKEEVFAQIDSSLSELLQERNTAPSSLLS

>AMK3 (Arabidopsis thaliana)

MATSSAASVDMEDIQTVDLMSELLRRMKCASKPDKRLVFIGPPGSGKGTQSPVIKDEFCLCHLSTGDMLRAAVAAKTPLGVKAKEAMDKGELVSDDLVVGIMDEAMNRPKCQKGFILDGFPRTVTQAEKLDEMLNRRGAQIDKVLNFAIDDSVLEERITGRWIHPSSGRSYHTKFAPPKVPGVDDLTGEPLIQRKDDNADVLRSRLDAFHKQTQPVIDYYAKKENLVNIPAEKAPEEVTKVVKKVVST

>AMK4 (Arabidopsis thaliana)

MATGGAAADLEDVQTVDLMSELLRRLKCSQKPDKRLIFIGPPGSGKGTQSPVVKDEYCLCHLSTGDMLRAAVASKTPLGVKAKEAMEKGELVSDDLVVGIIDEAMNKPKCQKGFILDGFPRTVTQAEKLDEMLKRRGTEIDKVLNFAIDDAILEERITGRWIHPSSGRSYHTKFAPPKTPGVDDITGEPLIQRKDDNADVLKSRLAAFHSQTQPVIDYYAKKAVLTNIQAEKAPQEVTSEVKKALS

>AMK5(Arabidopsis thaliana)

MASLSLSSAHFSSTSSSSRSSISTSSLSPSSTSLPLLQSPIRRRYRSLRRRLSFSVIPRRTSRSFSTSNSQIRCSINEPLKVMISGAPASGKGTQCELIVHKFGLVHISTGDLLRAEVSSGTDIGKRAKEFMNSGSLVPDEIVIAMVAGRLSREDAKEHGWLLDGFPRSFAQAQSLDKLNVKPDIFILLDVPDEILIDRCVGRRLDPVTGKIYHIKNYPPESDEIKARLVTRPDDTEEKVKARLQIYKQNSEAIISAYSDVMVKIDANRPKEVVFEETQTLLSQIQLKRMIKTDKASPVQDKWRGIPTRLNNIPHSRDIRAYFYEDVLQATIRSIKDGNTRLRVDINIPELNPEMDVYRIGTLMELVQALALSFADDGKRVKVCVQGSMGEGALAGMPLQLAGTRKILEYMDWGDDETLGTFVKLGAIGGKEVDEEDDMFILVAPQNAVGNCIIDDLQAMTTAAGKRPVVLINPRLKDLPASSGIMQTMGREQRLEYALTFDNCYVFRLLYYLGTQYPIMGALRMSYPYRYELYKRVNEENGKEKYVLLATYAERPTPEQIDDAFSGKSRDQSKKASGIWGFLSSVFS

>AMK6(Arabidopsis thaliana)

MAVSHRLLRPATTTIKNTFSSLFIRSLSSSSSGSSLDPKIDLEEAAAQLGKSSSTSTSPYKGRNFHWVFLGCPGVGKGTYASRLSSLLGVPHIATGDLVREELSSSGLLSSQLKELVNHGKLVPDEFIISLLSKRLQAGKDKGESGYILDGFPRTVTQAEILEGVTNIDLVINLKLREEALLAKCLGRRICSECGGNYNVACIDIKGDDDTPRMYMPPLLPPPNCESKLISRADDTEEVVKERLRIYNKMTQPVEEFYKKRGKLLEFELPGGIPESWARLLRALHLEDDKQSAIA//

>AMK7(Arabidopsis thaliana)

MAWLSRVRGVSPVTRLAAIRRSFGSAAALEFDYDSDDEYLYGDDRRLAEPRLGLDGSGPDRGVQWVLMGAPGAWRHVFAERLSKLLEVPHISMGSLVRQELNPRSSLYKEIASAVNERKLVPKSVVFALLSKRLEEGYARGETGFILHGIPRTRFQAETLDQIAQIDLVVNLKCSEDHLVNRNETALPQQEFLGSMLHSPVAINARRESVGVYAQEVEEYYRKQRKLLDFHVGGATSADTWQGLLAALHLKQVNLTTSQKLTL

>OsAK1(Oryza sativa)

MASSMAATATLSPPVLSAERPTVRGGLFLPPSPATSRSLRLQSARRCGISPATRKPRSLPRAAKVVVAVKADPLKVMIAGAPASGKGTQCELIKSKYGLVHISAGDLLRAEIAAGSENGKRAKEFMEKGQLVPDEIVVNMVKERLLQPDAQEKGWLLDGYPRSYSQAMALETLNIRPDIFILLDVPDELLVERVVGRRLDPVTGKIYHLKYSPPENEEIASRLTQRFDDTEEKVKLRLQTHYQNVESLLSIYEDVIVEVKGDALVDDVFAEIDKQLTSSLDKKTEMVASA

>OsAK3 (Oryza sativa)

MAANLEDVPSMELMTELLRRMKCSSKPDKRVILVGPPGCGKGTQSPLIKDEFCLCHLATGDMLRAAVAAKTPLGIKAKEAMDKGELVSDDLVVGIIDEAMKKTSCQKGFILDGFPRTVVQAQKLDEMLAKQGTKIDKVLNFAIDDAILEERITGRWIHPSSGRSYHTKFAPPKTPGLDDVTGEPLIQRKDDTAAVLKSRLEAFHVQTKPVIDYYTKKGIVANLHAEKPPKEVTVEVQKALS

>OsAK4 (Oryza sativa)

MAAAANLEDVPSMDLMNELLRRMKCSSKPDKRLILVGPPGSGKGTQSPIIKDEYCLCHLATGDMLRAAVAAKTPLGVKAKEAMDKGELVSDDLVVGIIDEAMKKPSCQKGFILDGFPRTVVQAQKLDEMLEKKGTKVDKVLNFAIDDSILEERITGRWIHPSSGRSYHTKFAPPKVPGVDDVTGEPLIQRKDDTAEVLKSRLEAFHKQTEPVIDYYSKKALVANLHAEKPPKEVTAEVQKVLS
