# Supplementary material for: An Adenylate Kinase OsAK3 Involves Brassinosteroid Signaling and Grain Length in Rice (Oryza sativa L.)
Source: Rice (N Y). 2021 Dec 28;14:105. doi: 10.1186/s12284-021-00546-0 (PMC8714616; doi:10.1186/s12284-021-00546-0)
Supplement: Supplementary file 2 — Additional file 2: Fig. S1. Colorimetric quantitation of OsAK3 activity in the presence of qGL3 in vitro. Fig. S2. OsAK4 has the highest homology with OsAK3 and interacts with qGL3. Fig. S3. Genotyping analysis of osak3 and osak3-r mutant. Fig. S4. Morphological characteristics of osak3-r mutant. Fig. S5. Effects of BL treatment in osak3-r. Fig. S6. Effects of BL treatment in osak3. Fig. S7. Effects of BL treatment in OsAK3-OXs plants. Fig. S8. RT-qPCR verification of DEGs selected from RNA-seq. Fig. S9. Overview of the DEGs between DJ and osak3 mutant. Fig. S10. OsAK3 is involved in multiple phytohormone signaling pathways and stress responses. Fig. S11. A working model for the functions of OsAK3 in BR signaling and plant growth and development. [file 12284_2021_546_MOESM2_ESM.docx]

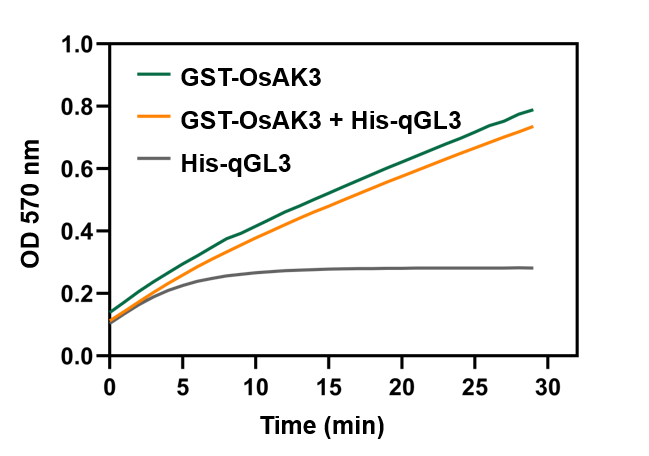


**Fig. S1** Colorimetric quantitation of OsAK3 activity in the presence of qGL3 *in vitro*.


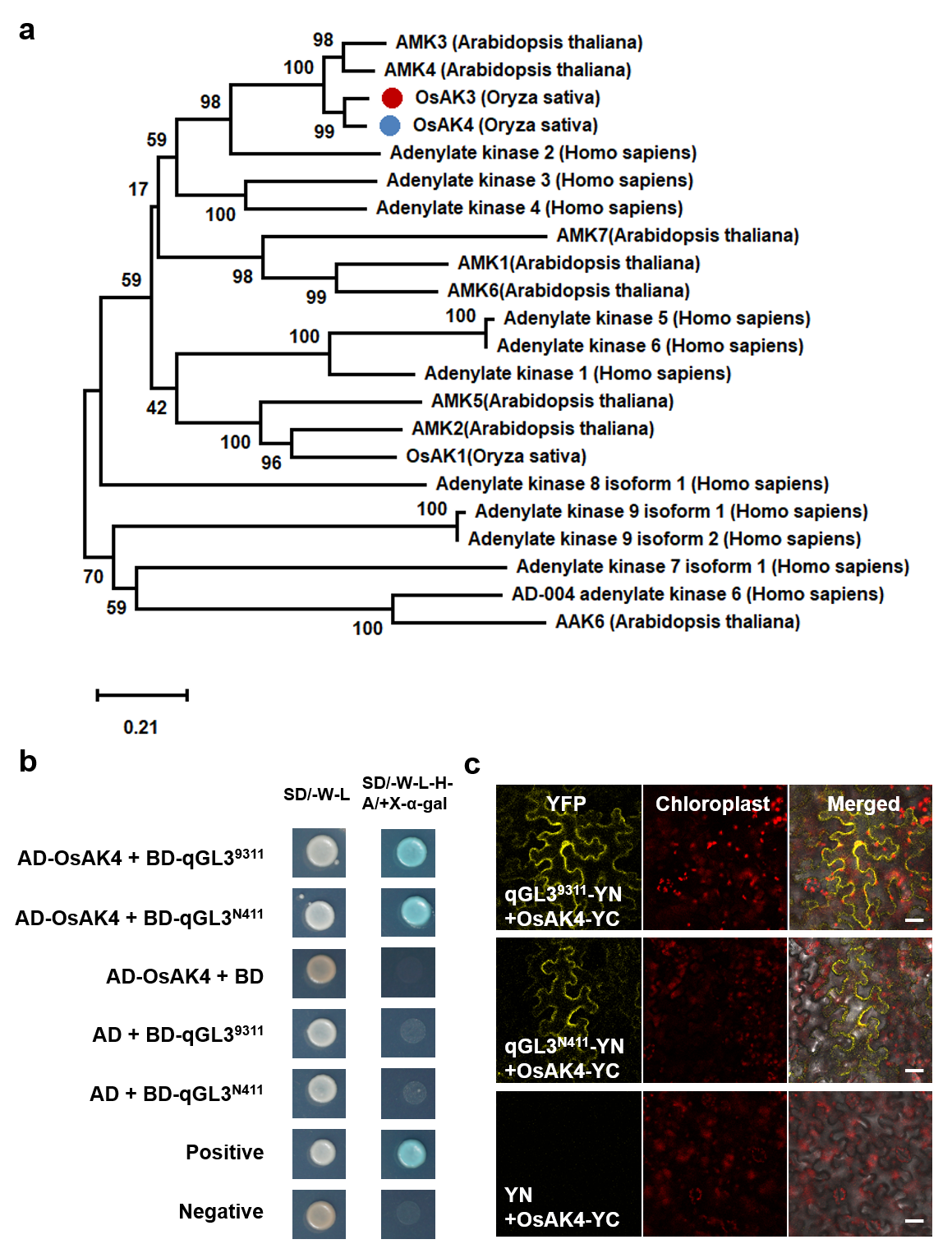
**Fig. S2** OsAK4 has the highest homology with OsAK3 and interacts with qGL3. **a** Phylogenetic analysis of adenylate kinases from human, Arabidopsis and rice. **b** Y2H analysis of the interaction between OsAK4 and qGL3. **c** BiFC analysis of the interaction between OsAK4 and qGL3 (scale bars, 50 μm).


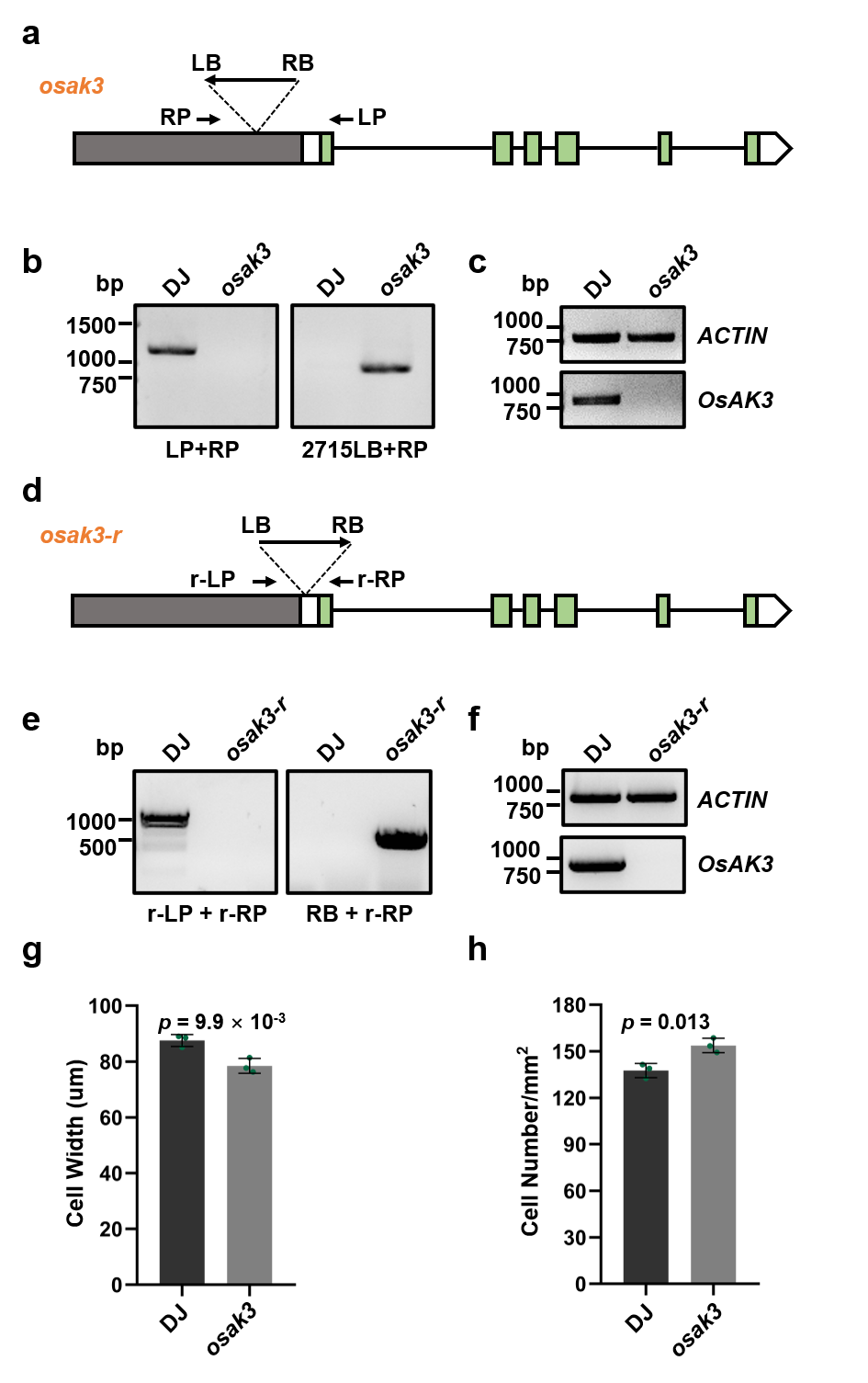


**Fig. S3** Genotyping analysis of *osak3* and *osak3-r* mutant. **a** T-DNA insertion site in *osak3* mutant. Green boxes indicate exons, white boxes indicate UTRs, grey box indicate promotor, and lines between boxes are introns. **b** PCR verification of the insertion site in *osak3* mutant. **c** Expression analysis of *OsAK3* in DJ and *osak3* mutant by RT-sqPCR strategy. **d** T-DNA insertion site in *osak3-r* mutant. **e** PCR verification of the insertion site in *osak3-r* mutant. **f** Expression analysis of *OsAK3* in DJ and *osak3-r* mutant by RT-sqPCR strategy. **g, h** Statistical data of spikelet glume cell width (**g**) and cell number (**h**) of *osak3* mutant. Data are means ± _SD_ (*n* = 3). P value compared with the wild type by student’s *t*-test.
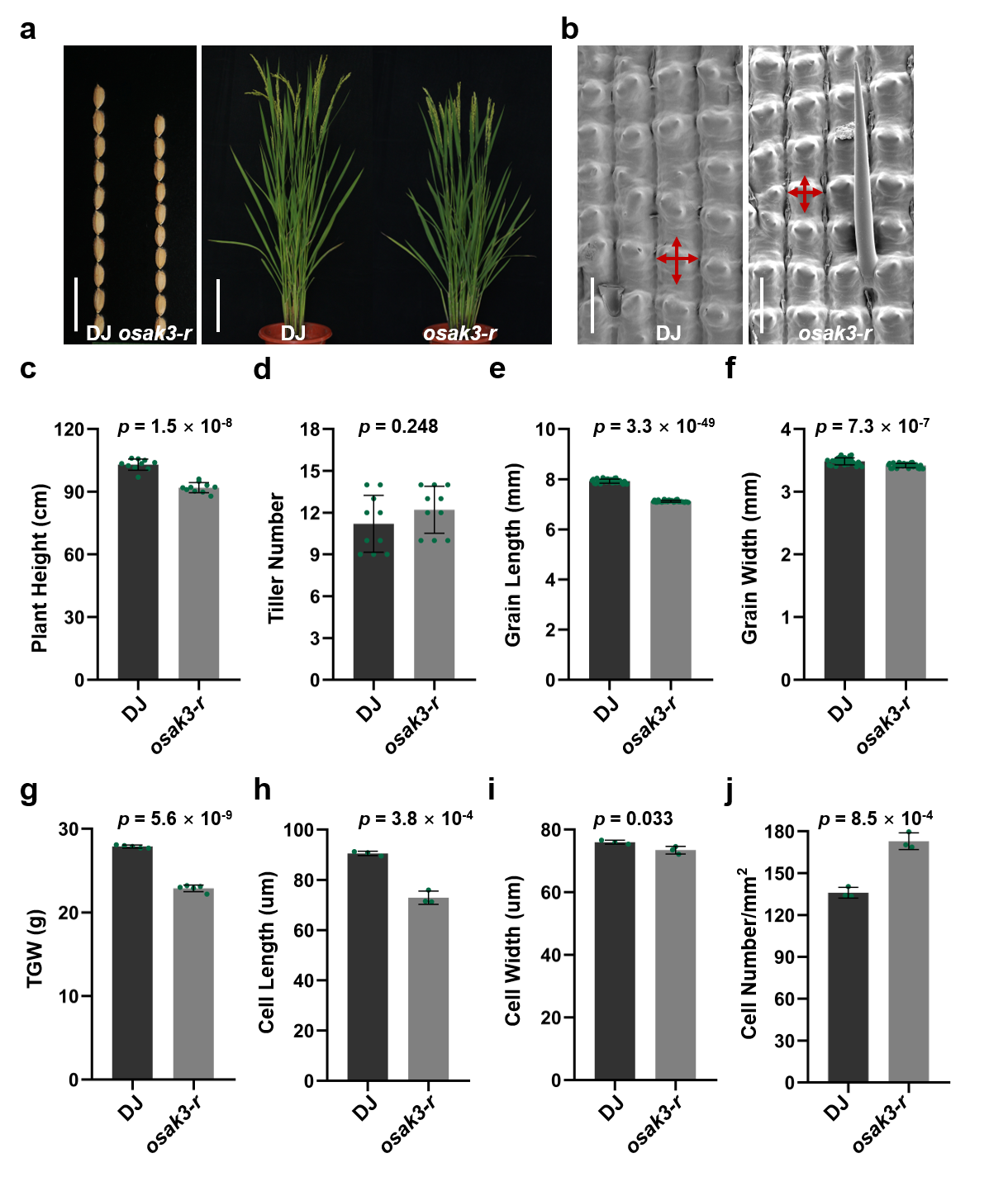


**Fig. S4** Morphological characteristics of *osak3-r* mutant. **a** Plant phenotype of DJ and *osak3-r* at the mature stage. From left to right are grain size (scale bar, 1 cm) and plant profile (scale bar, 20 cm). **b** Scanning electron microscopic observation of the spikelet glume cells of DJ and *osak3-r*. Scale bars, 100 μm. **c, d** Statistical data of plant height (**c**) and tiller number (**d**). Data are means ± _SD_ (*n* = 10). P value compared with the wild type by student’s t-test. **e, f** Statistical data of grain length (**e**) and grain width (**f**). Data are means ± _SD_ (*n* = 30). P value compared with the wild type by student’s *t*-test. **g** Statistical data of 1,000-grain weight. Data are means ± _SD_ (*n* = 5). P value compared with the wild type by student’s *t*-test. **h-j** Statistical data of spikelet glume cell length (**h**), cell width (**I**) and cell number (**j**). Data are means ± _SD_ (*n* = 3). P value compared with the wild type by student’s *t*-test.


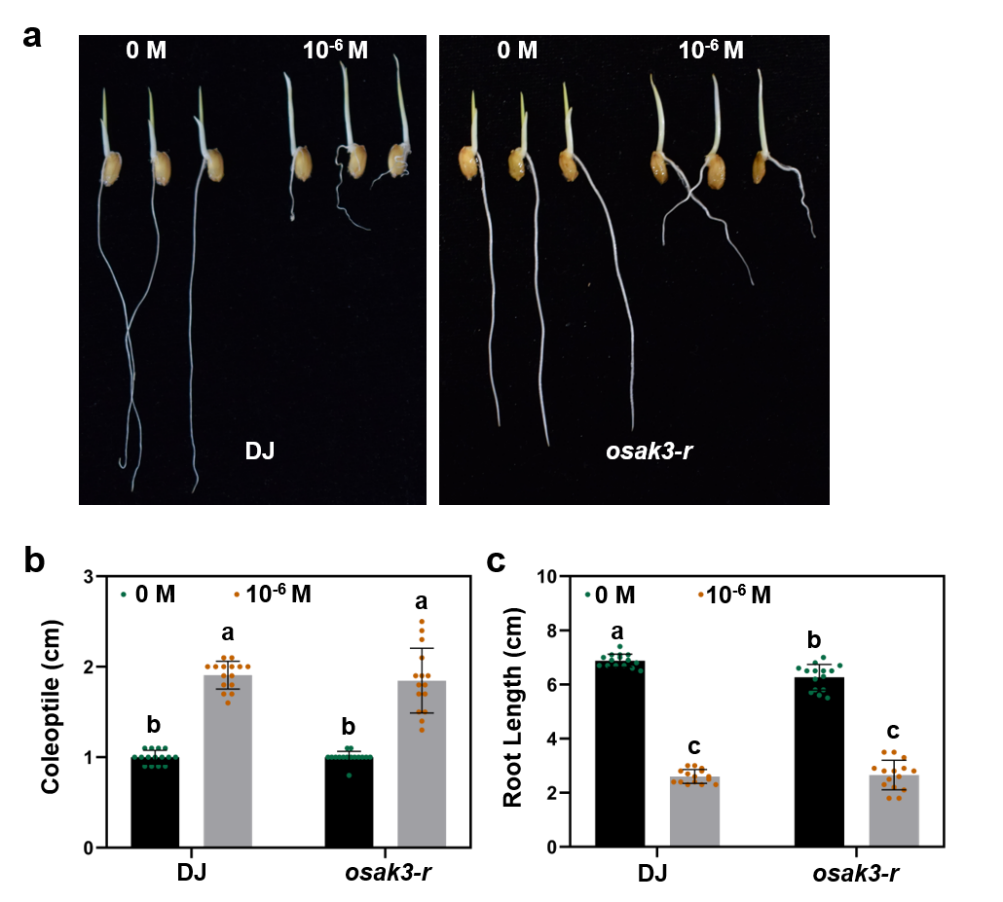


**Fig. S5** Effects of BL treatment in *osak3-r*. **a** Coleoptile elongation analysis and root inhibition analysis of DJ and *osak3-r* mutant in response to 0 M, 10^-8^ M,10^-7^ M and 10^-6^ M BL. **b** Statistical data of coleoptile elongation analysis in DJ and *osak3-r* mutant. Data are means ± _SD_ (*n* = 15). Statistical analyses were performed by Duncan’s multiple range tests. The presence of the same lowercase letter denotes a non-significant difference between means (P>0.05). **c** Statistical data of root inhibition analysis in DJ and *osak3-r* mutant. Data are means ± _SD_ (*n* = 15). Statistical analyses were performed by Duncan’s multiple range tests. The presence of the same lowercase letter denotes a non-significant difference between means (P>0.05).


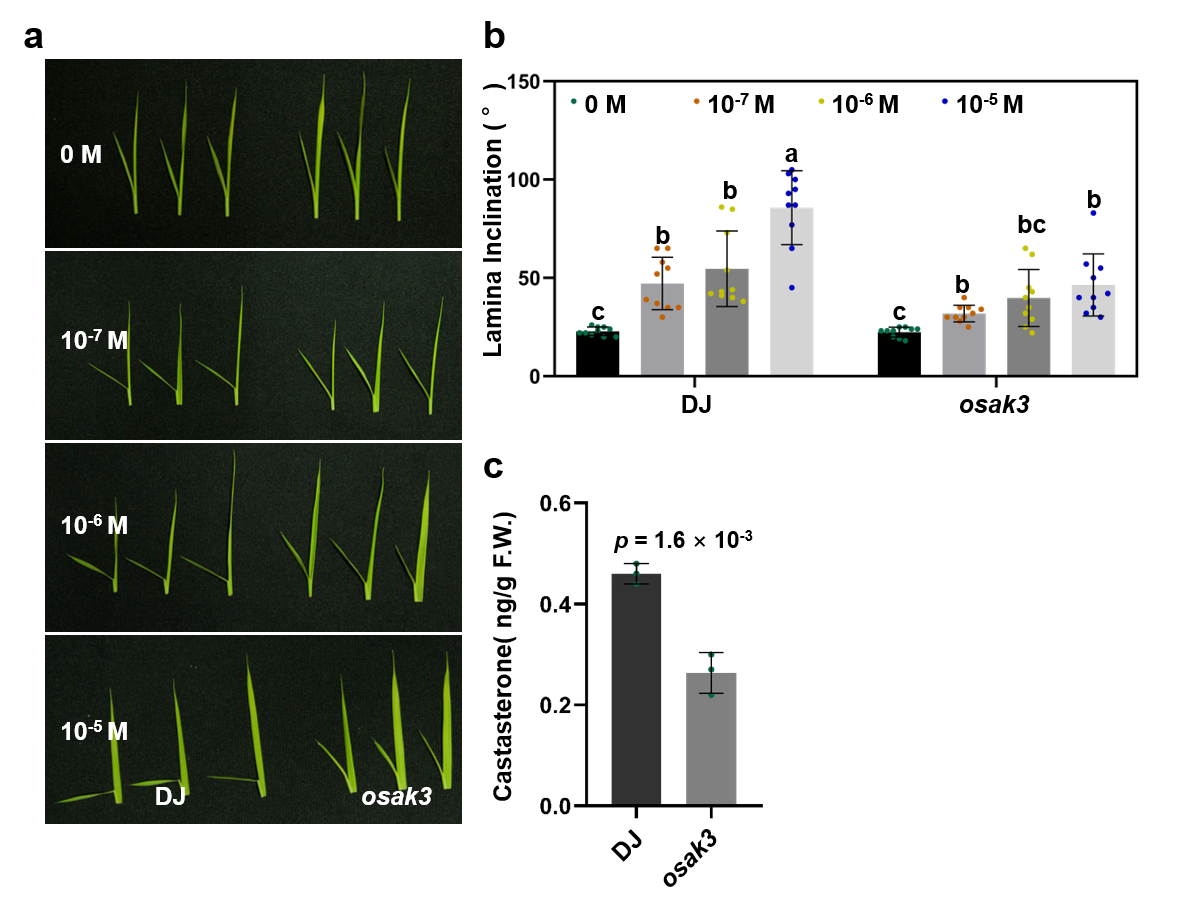
**Fig. S6** Effects of BL treatment in *osak3*. **a** Lamina inclination assay of DJ and *osak3* mutant in response to 0 M, 10^-7^ M,10^-6^ M and 10^-5^ M BL. **b** Statistical data of lamina inclination assay in DJ and *osak3* mutant. Data are means ± _SD_ (*n* =10). Statistical analyses were performed by Duncan’s multiple range tests. The presence of the same lowercase letter denotes a non-significant difference between means (P>0.05). **c** The content of Castasterone in DJ and *osak3*. Data are means ± _SD_ (*n* = 3). P value compared with the wild type by student’s *t*-test.

**
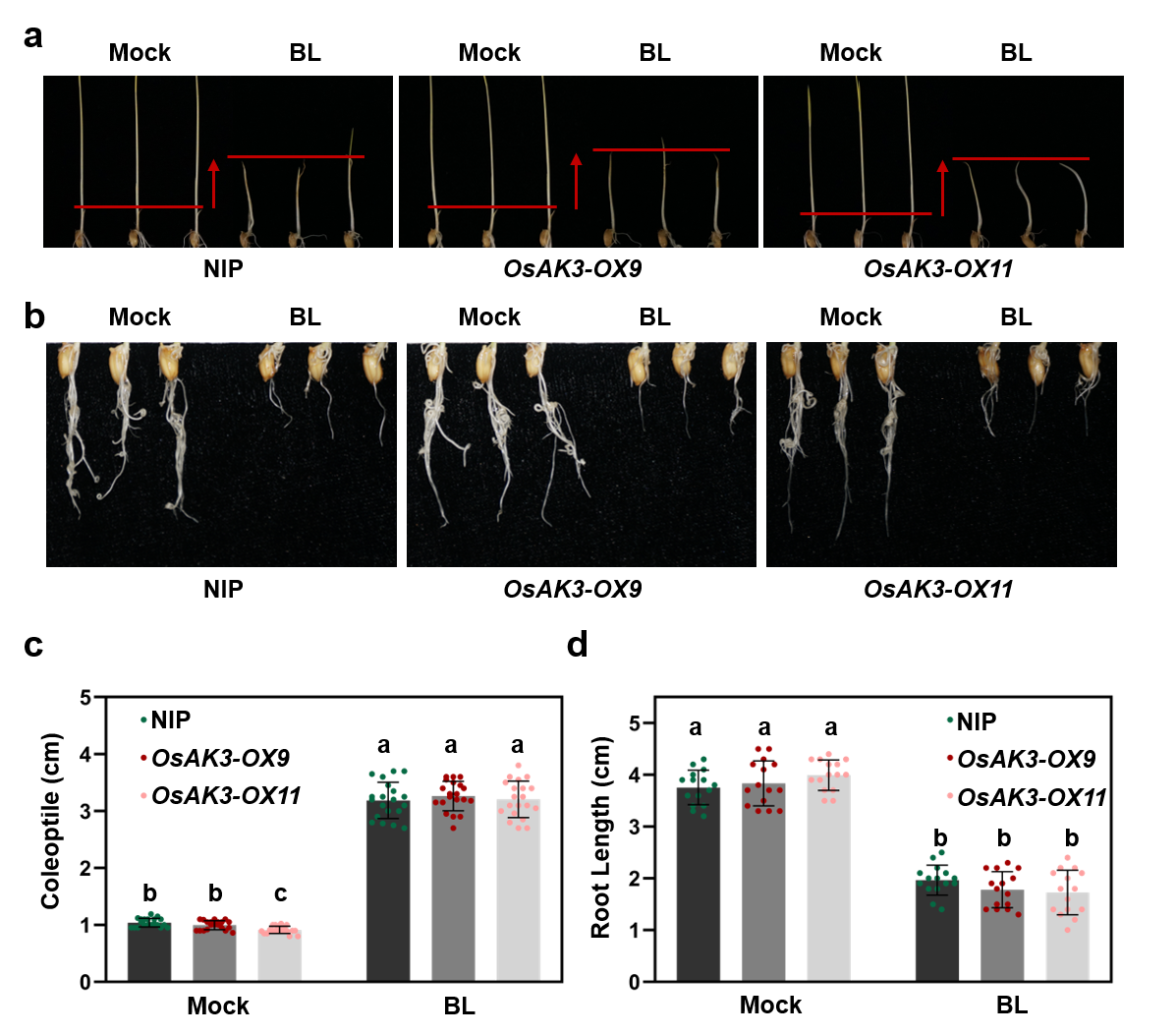
Fig. S7** Effects of BL treatment in *OsAK3-OXs* plants. **a** Coleoptile elongation analysis of NIP and *OsAK3-OXs* plants in response to 10^-6^ M BL. The red line represents the top of the coleoptile and the red arrow indicates the increase in coleoptile length. **b** Root inhibition analysis of NIP and *OsAK3-OXs* plants in response to 10^-6^ M BL. **c** Statistical data of coleoptile elongation analysis in NIP and *OsAK3-OXs* plants using 10^-6^ M BL. Data are means ± _SD_ (*n* = 20). Statistical analyses were performed by Duncan’s multiple range tests. The presence of the same lowercase letter denotes a non-significant difference between means (P>0.05). **d** Statistical data of root inhibition analysis in NIP and *OsAK3-OXs* plants using 10^-6^ M BL. Data are means ± _SD_ (*n* = 15). Statistical analyses were performed by Duncan’s multiple range tests. The presence of the same lowercase letter denotes a non-significant difference between means (P>0.05).


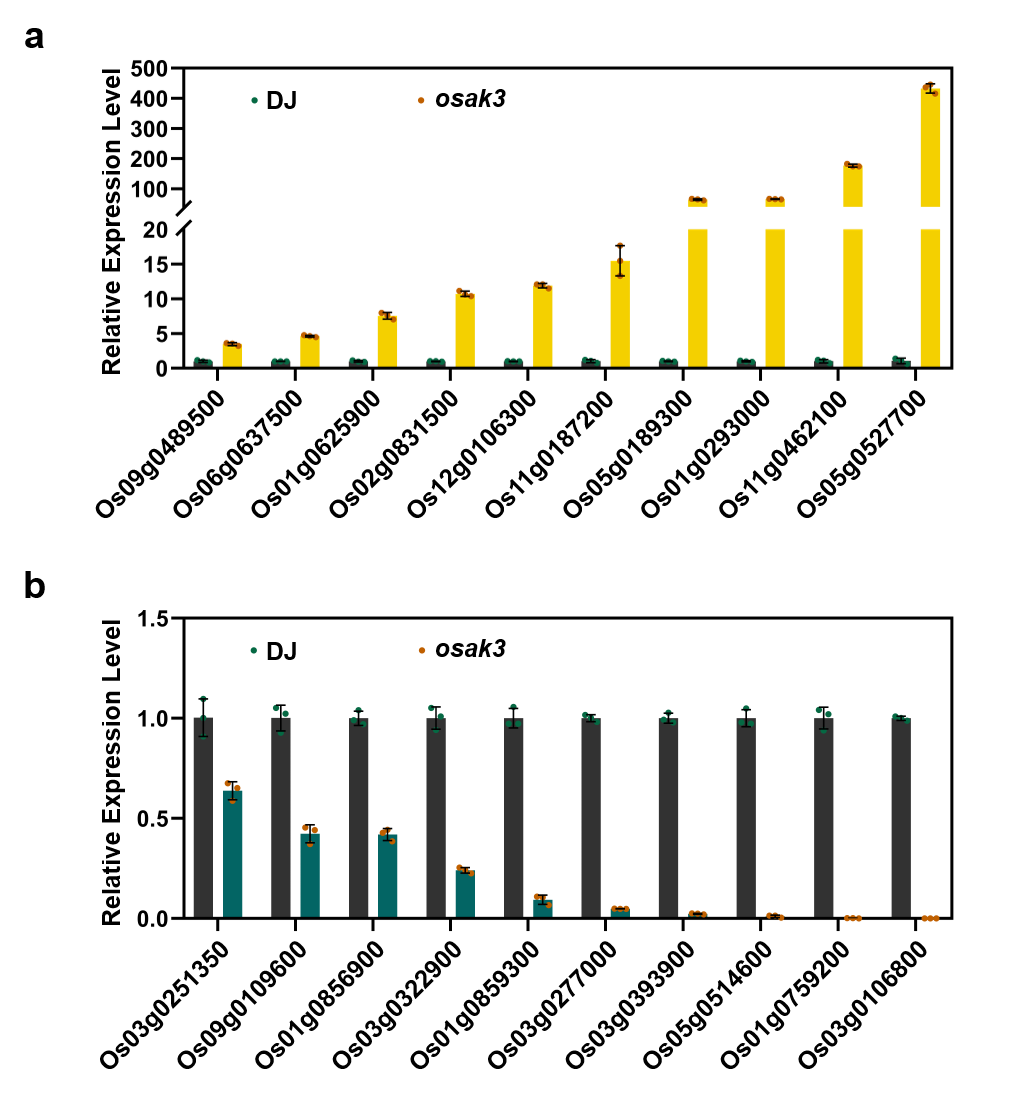
**Fig. S8** RT-qPCR verification of DEGs selected from RNA-seq. **a, b** RT-qPCR validation of up-regulated (**a**) and down-regulated (**b**) DEGs. Data are means ± _SD_ (*n* = 3).

**Fig. S9** Overview of the DEGs between DJ and *osak3* mutant. **a** Metabolism overview of the DEGs between DJ and *osak3* mutant. **b** Regulation overview of the DEGs between DJ and *osak3* mutant. **c** Cell function overview of the DEGs between DJ and *osak3* mutant. **d** Biotic stress overview of the DEGs between DJ and *osak3* mutant. **e** Cellular response overview of the DEGs between DJ and *osak3* mutant. The images were obtained using MapMan. Red, up-regulation; white, no-difference; blue, down-regulation.


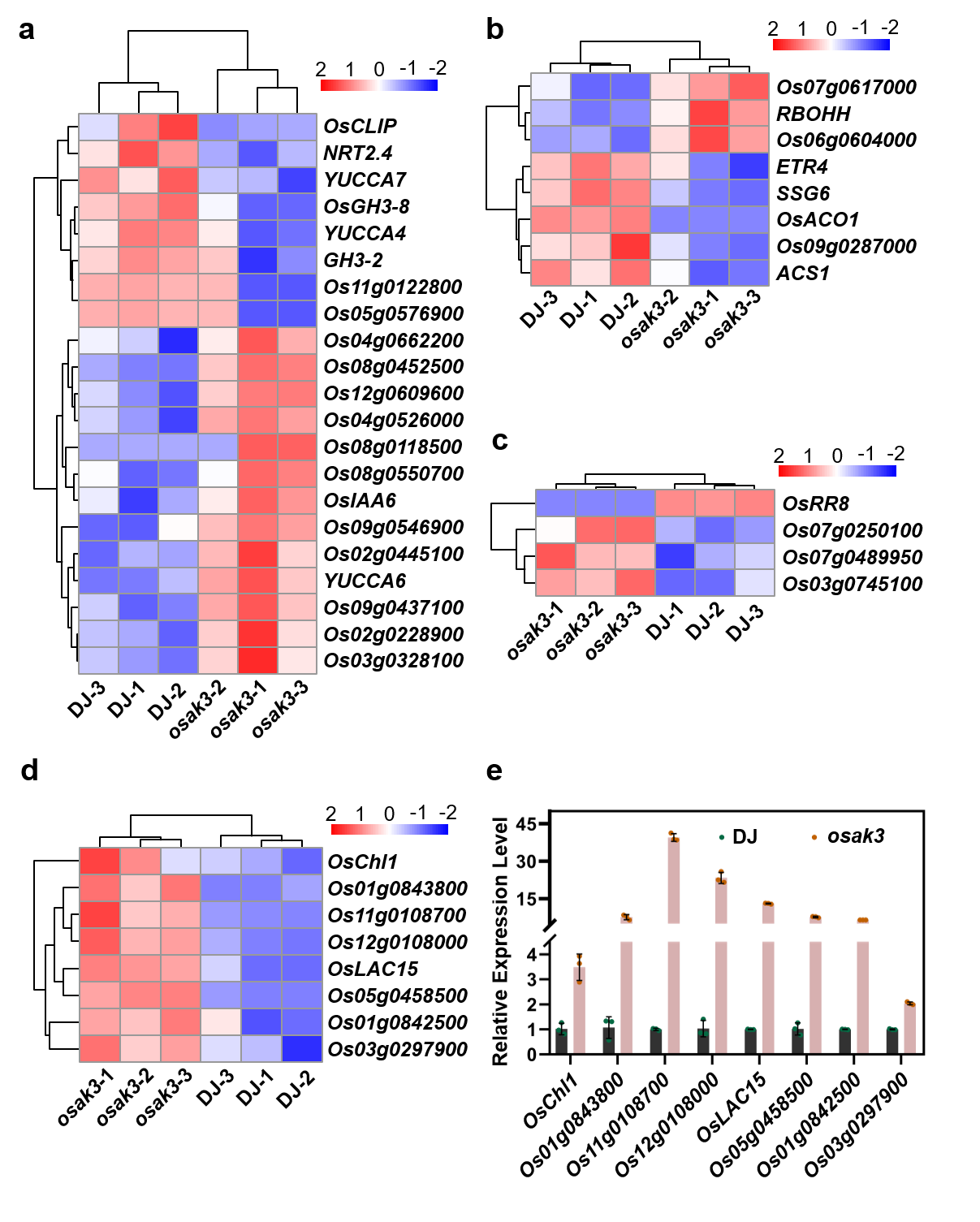
**Fig. S10** *OsAK3* is involved in multiple phytohormone signaling pathways and stress responses. **a-c** Heat map of the DEGs related to auxin (**a**), ethylene (**b**), and cytokinin (**c**). Scale bar shows fold changes, values are normalized by z-score scheme, red and blue color indicate up- and down-regulated, respectively. **d** Heat map of the DEGs annotated as laccase related. Scale bar shows fold changes, values are normalized by z-score scheme, red and blue color indicate up- and down-regulated, respectively. **e** RT-qPCR validation of DEGs in (**e**). Data are means ± _SD_ (*n* = 3).


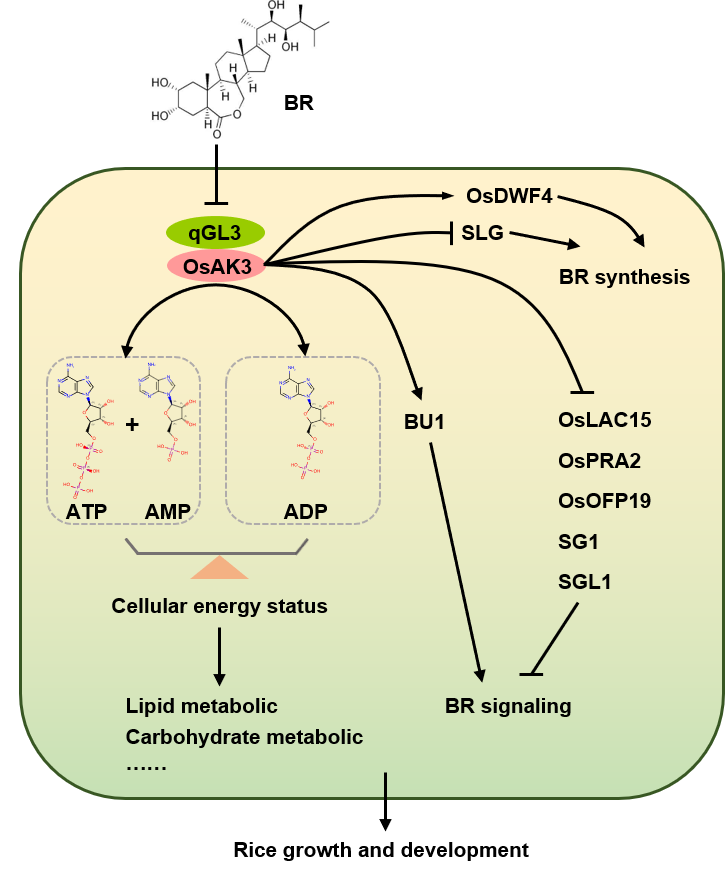
**Fig. S11** A working model for the functions of OsAK3 in BR signaling and plant growth and development. OsAK3 catalyzes a reversible transphosphorylation reaction that converts ADP to ATP and AMP. Adenylate energy charge (AEC) ratio affects cellular energy status, which in turn alters energy-related metabolic processes. BR signaling induces *OsAK3* expression, and mutation in *OsAK3* affects the expression of BR-responsive genes and grain length related genes, further regulating the growth and development of rice. Genes listed in this model are DEGs screened from RNA-seq.

**Table S1.** Primers used in this study.

**Table S2.** List of DEGs used for RT-qPCR validation.

**Table S3.** List of DEGs associated with floral organ development detected in DJ and *osak3*.

**Table S4.** List of DEGs associated with stress response detected in DJ and *osak3*.

**Supplemental Data Set 1.** Adenylate kinase sequences used in phylogenetic tree analysis.
